# RNAseq profiling of blood from patients with coronary artery disease: Signature of a T cell imbalance

**DOI:** 10.1016/j.jmccpl.2023.100033

**Published:** 2023-03-25

**Authors:** Timothy A. McCaffrey, Ian Toma, Zhaoqing Yang, Richard Katz, Jonathan Reiner, Ramesh Mazhari, Palak Shah, Zachary Falk, Richard Wargowsky, Jennifer Goldman, Dan Jones, Dmitry Shtokalo, Denis Antonets, Tisha Jepson, Anastasia Fetisova, Kevin Jaatinen, Natalia Ree, Maxim Ri

**Affiliations:** a Department of Medicine, Division of Genomic Medicine, The George Washington University, 2300 I Street NW, Washington, DC 20037, United States of America; b Department of Medicine, Division of Cardiology, The George Washington University, 2300 I Street NW, Washington, DC 20037, United States of America; c SeqLL, Inc., 3 Federal Street, Billerica, MA 01821, United States of America; d The St. Laurent Institute, 317 New Boston Street, Woburn, MA 01801, United States of America; e Department of Microbiology, Immunology, and Tropical Medicine, The George Washington University, 2300 I Street NW, Washington, DC 20037, United States of America; f Department of Clinical Research and Leadership, The George Washington University, 2300 I Street NW, Washington, DC 20037, United States of America; g A.P. Ershov Institute of Informatics Systems SB RAS, 6, Acad. Lavrentyeva Ave, Novosibirsk 630090, Russia; h INOVA Heart and Vascular Institute, 3300 Gallows Road, Fairfax, VA 22042, United States of America; i True Bearing Diagnostics, 2450 Virginia Avenue, Washington, DC 20037, United States of America; j Center for Mitochondrial Functional Genomics, Institute of Living Systems, Immanuel Kant Baltic Federal University, Kalingrad 236040, Russia

**Keywords:** Atherosclerosis, Transcriptome, RNA sequencing, Regulatory T cells, Treg, Network analysis, Coronary artery disease, Cilia, Immune synapse

## Abstract

**Background:**

Cardiovascular disease had a global prevalence of 523 million cases and 18.6 million deaths in 2019. The current standard for diagnosing coronary artery disease (CAD) is coronary angiography either by invasive catheterization (ICA) or computed tomography (CTA). Prior studies employed single-molecule, amplification-independent RNA sequencing of whole blood to identify an RNA signature in patients with angiographically confirmed CAD. The present studies employed Illumina RNAseq and network co-expression analysis to identify systematic changes underlying CAD.

**Methods:**

Whole blood RNA was depleted of ribosomal RNA (rRNA) and analyzed by Illumina total RNA sequencing (RNAseq) to identify transcripts associated with CAD in 177 patients presenting for elective invasive coronary catheterization. The resulting transcript counts were compared between groups to identify differentially expressed genes (DEGs) and to identify patterns of changes through whole genome co-expression network analysis (WGCNA).

**Results:**

The correlation between Illumina amplified RNAseq and the prior SeqLL unamplified RNAseq was quite strong (r = 0.87), but there was only 9 % overlap in the DEGs identified. Consistent with the prior RNAseq, the majority (93 %) of DEGs were down-regulated ∼1.7-fold in patients with moderate to severe CAD (>20 % stenosis). DEGs were predominantly related to T cells, consistent with known reductions in Tregs in CAD. Network analysis did not identify pre-existing modules with a strong association with CAD, but patterns of T cell dysregulation were evident. DEGs were enriched for transcripts associated with ciliary and synaptic transcripts, consistent with changes in the immune synapse of developing T cells.

**Conclusions:**

These studies confirm and extend a novel mRNA signature of a Treg-like defect in CAD. The pattern of changes is consistent with stress-related changes in the maturation of T and Treg cells, possibly due to changes in the immune synapse.

## Highlights

• The largest RNAseq analysis of blood RNA from patients with angiographically confirmed coronary artery disease (CAD).
• RNAseq was performed at high depth on the Illumina amplified sequencing platform and compared with SeqLL single molecule sequencing.
• Both platforms identified transcripts that were of low to modest abundance, but almost exclusively decreased in CAD patients.
• Cell type analysis suggest they are related to an altered state of T cells, especially T regulatory cells.
• These studies provide a genomic-scale expression map and a resource for defining transcriptome changes related to CAD.

## Graphical abstract

**Figure:**
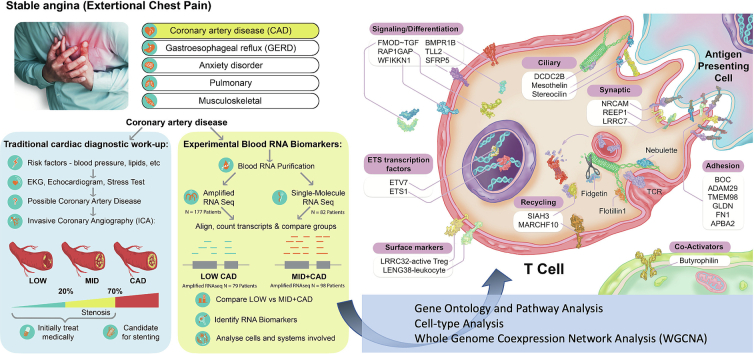

## Abbreviations

CAD: coronary artery disease;
CBC: complete blood count
CPC: circulating progenitor cells
ddPCR: droplet digital PCR
DEGs: differentially expressed genes
GERD: gastroesophageal reflux disease
ICA: invasive coronary angiography
RIN: RNA integrity number
RNAseq: RNA sequencing
rRNA: ribosomal RNA
RPKM: reads per kilobase of exon per million mapped total reads
STEMI: ST segment elevation myocardial infarction
SLE: systemic lupus erythematosus
tSMS: true single molecule sequencing
TRACs: transcripts associated with CAD
Treg: regulatory T cell

## 1 Introduction

Roughly 2200 Americans die of cardiovascular disease each day, about one person every 40 s, with more than a million heart attacks each year [1]. The classic symptoms of coronary artery disease (CAD) are chest pain and shortness of breath upon exertion. However, these clinical signs alone are neither sensitive nor specific for CAD. Most chest pain cases are due to musculoskeletal causes (20 %) or gastroesophageal reflux disease (GERD) (13 %), while CAD is the cause in only 11 % of cases, with minor contributions from pulmonary, neurological, or idiopathic causes [2], [3]. The clinical risk factors of advancing age, such as male sex, elevated cholesterol, smoking, and hypertension, are good predictors of long term risk (30 yr. risk, C statistic = 0.803) [4], however, they are less useful in acute clinical settings for determining whether a person has CAD (C statistic = 0.667, where 0.5 is random chance) [5].

Thus, the diagnosis of CAD would benefit from additional diagnostic tools to assess the need for coronary imaging or intervention. Each year, there are more than one million cardiac catheterizations, of which 622,000 result in interventions such as stent placement [6]. Using the best clinical prediction models that combine symptoms and non-invasive testing, it remains surprising that 20–40 % of angiograms performed ultimately do not detect any occluded arteries [5], [7], [8], [9], [10]. Prior analysis of registries examining almost 400,000 patients, identified about 40 % of patients undergoing invasive coronary angiography (ICA) as having <20 % stenosis [5]. The increasingly conservative use of ICA, and the advent of computed tomography angiography (CTA) will likely reduce the rate of normal ICAs. However, there is little question that reliable blood-based biomarkers of CAD would have the potential to further reduce the number of cardiac catheterizations on relatively low risk individuals, as well as potentially detecting asymptomatic CAD.

Prior studies have utilized microarray-based approaches to identify transcripts in blood that are related to CAD, and derived transcript subsets have even reached some clinical practice [11], [12], [13], [14], [15]. Overall, however, these different microarray-based studies identified different RNA biomarkers for CAD, and the biomarkers brought to the clinic incorporated patient age and sex to achieve modest predictive ability [16]. A recurring problem with microarrays, when applied to blood RNA, is relatively high noise created by very abundant transcripts, such as globins. Abundant RNAs can overwhelm the detection of changes of low magnitude, or larger changes that occur in only a small subset of cells [17]. Thus, our prior studies employed the SeqLL single-molecule RNA sequencing (RNAseq) methodology to identify a novel set of transcripts associated with the presence of CAD, but essentially unrelated to other known risks for CAD [18]. The pattern of changes was consistent with a large literature implicating a role of regulatory T cell (Treg) dysfunction as an important component in the etiology of CAD [19], [20]. The present studies compared the single-molecule, unamplified SeqLL RNAseq to amplification-based Illumina Total RNAseq in 82 patients, and then expanded the cohort to 177 patients with angiographically confirmed CAD. Extensive analysis of the differentially expressed genes confirmed a T-cell related pattern of changes in CAD, but with clear differences in the specific transcripts associated with CAD depending on the RNAseq platform employed.

## 2 Methods

### 2.1 Experimental design

**Fig. 1:**
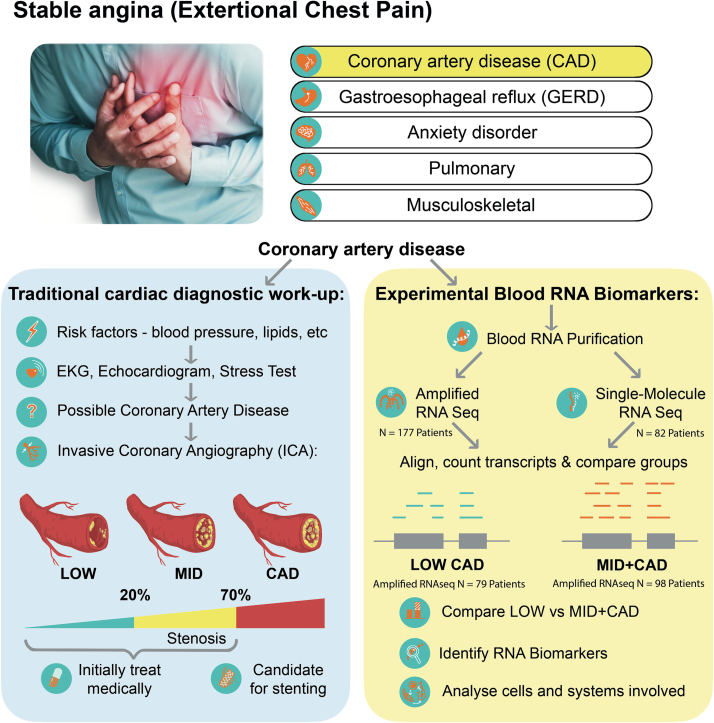
Schematic of study design. Patients presenting to their cardiologist with stable chest pain can have several underlying disorders, including CAD. The patients referred for invasive coronary angiography (ICA) were candidates for the current study. On the basis of ICA results, patients were categorized into LOW (stenosis <20 %, N = 79), or MID + CAD (stenosis >20 %, N = 98) groups (left panel). Prior to ICA, a blood sample (3 ml) was drawn into Tempus blood RNA stabilizer, and frozen at −80 °C until it was analyzed by Illumina Total RNAseq (amplified). A subset of 82 patients was also analyzed by SeqLL true single-molecule (tSMS, unamplified) RNAseq. Differentially expressed transcripts were identified and analyzed as described (right panel).

Invasive coronary angiography (ICA) was used to identify patients with obstructive CAD versus those without CAD (Fig. 1). Blood was collected in Tempus RNA stabilizing tubes prior to ICA, stored at −80 °C and then profiled by RNAseq as described below. Coronary angiograms were digitally interpreted by the attending cardiologist, and the patients were divided into 3 groups, <20 % stenosis (LOW CAD), >20 % but <70 % stenosis of any vessel (MID CAD), and >70 % stenosis of any artery (CAD). For power and simplicity, initial analyses compared LOW CAD (<20 % stenosis) to MID and CAD combined (>20 % stenosis) yielding groups of similar size.

### 2.2 Patients

#### 2.2.1 Discovery cohort

Patients presenting for non-emergent complaints of typical or atypical chest pain, exertional dyspnea, or other symptoms suggestive of CAD provided written, informed consent for participation in this study under a protocol approved by the George Washington University IRB and the INOVA Fairfax IRB. Patients with heart failure, non-ST segment elevation myocardial infarction (MI) and ST elevation MI (STEMI) were excluded from the study. The design of the study is shown schematically in Fig. 1. Patients admitted for diagnostic cardiac catheterization had three Tempus blood RNA tubes collected by peripheral venipuncture or an indwelling catheter. After blood sampling, these studies were purely observational and did not alter in any way the patient's clinical course. All relevant clinical data, including a complete blood count (CBC), was captured for comparison to the transcriptomic studies. From an initial enrollment of 85 patients, 82 patients had complete clinical and RNA sequencing data for further analysis.

#### 2.2.2 Validation cohort

A subsequent, independent group of patients drawn from the same ongoing study were consented at INOVA Fairfax Hospital (Fairfax, Virginia) who were likewise undergoing routine, elective ICA for evaluation of suspected CAD. A total of 95 patients had sufficiently complete clinical and RNAseq data for further analysis.

### 2.3 Clinical risk factor assessment

Cardiac medical histories were evaluated by their attending cardiologists to determine CAD risk factors, according to the 2013 ACC/AHA Guidelines on the Assessment of Cardiovascular Risk [21]. Hypertension was indicated by a history of blood pressure ≥ 140/90 mmHg and/or treatment with anti-hypertensive medications. A family history of CAD was determined by MI or cardiac death in a first-degree relative. Diabetes mellitus was indicated by fasting glucose of ≥126 mg/dl and/or use of insulin or oral hypoglycemic agents. Current smoking was defined by active smoking within the most recent 3 months. Dyslipidemia was adjudicated by the guidelines of National Cholesterol Education Program Adult Treatment Panel III or by treatment with lipid lowering medication.

### 2.4 Transcriptome profiling

#### 2.4.1 RNA processing

Tempus stabilized frozen (−80 °C) peripheral blood samples were thawed and RNA was isolated using Tempus Spin RNA Isolation Kits (ThermoFisher Scientific) according to the manufacturer's protocol. The total nucleic acid isolate was treated with 4 Units of DNAse (Turbo DNA-free Kit, Ambion). The typical nucleic acid yield from 3 ml Tempus blood tubes averaged ∼3 μg, with an RNA integrity (RIN) score > 7 (10 is maximal) on Agilent 2100 Bioanalyzer (Table 1).

**Table 1:** Demographic and analytical characteristics of the cohort.

	LOW		CAD			P value
	Mean	(sem)	Mean	(sem)		
N	79		98			
Sex (%Male)	59.5 %		65.3 %			0.427
Age (yrs)	63.60	1.18	69.18	1.16	⁎	0.001
Race (%White)	79.7 %		85.7 %			0.293
Ethnicity (%non-Hisp)	92.4 %		92.9 %			0.909
Stenosis (%Lumen)	2.95	0.57	67.55	2.66	⁎	<0.0005
Ejection Fraction (%)	55.68	1.19	57.47	1.07		0.266
Body Mass Index (kg/m sq)	31.49	1.06	30.02	0.76		0.252
Systolic BP (mm Hg)	129.10	2.05	137.58	2.59	⁎	0.014
Diastolic BP (mm Hg)	71.78	1.25	73.70	1.30		0.296
Heart Rate (BPM)	76.67	2.78	79.15	3.14		0.564
Dyslipidemia (%)	45.6 %		60.2 %			0.052
Hypertension (%)	48.1 %		70.4 %		⁎	0.003
Diabetes mellitus (%)	21.5 %		31.6 %			0.132
Family history of CAD (%)	41.8 %		30.6 %			0.123
Smoking (%)	64.6 %		52.0 %			0.094
ASA (mg/day)	37.95	5.82	69.47	6.45	⁎	<0.0005
Creatinine (mg/dL)	0.96	0.03	1.05	0.04		0.053
Blood RNA Yield (ug/3 ml)	3.16	0.09	3.24	0.09		0.529
RNA Integrity (1−10)	7.38	0.09	7.31	0.08		0.592
Raw Reads/patient (M)	25.6	0.84	24.8	0.76		0.462
Filtered Reads/patient (M)	18.3	0.58	17.7	0.56		0.459
Reads/Patient (M)	14.7	0.45	14.4	0.47		0.622
Reads %/patient	81.0 %	0.83 %	81.1 %	0.63 %		0.939

# Discrete variables (sex, race, ethnicity, dyslipidemia, hypertension, diabetes, family history, and smoking were compared by a chi-square test), all others were compared by Student's *t-*test.

⁎ Indicates p < 0.05 uncorrected for multiple testing.

#### 2.4.2 RNA sequencing

The total RNA, post-DNAse, was sequenced using the Illumina TruSeq Stranded Total RNA sequencing kit, which includes depletion of ribosomal RNA (rRNA) by Ribo-Zero rRNA Removal Kit (Illumina). Each RNAseq run was composed of 24 patient blood RNAs barcoded for multiplexing onto the NextSeq 500 using the High-Output 2x75bp kit. The resulting 150 bp paired end reads were parsed to each barcode/patient, concatenated across the 4 read chambers, trimmed, and then aligned to the HG38 genome [22] using standard R packages in Galaxy [23]. The analytical flow is summarized in Sup. Data 1 and the Galaxy script is available with the GEO data. The number of reads that align to each transcript in a 28 K gene level transcriptome was counted. For an RPKM filtering analysis, similar to our published SeqLL tSMS data [18], the raw read count was adjusted by the size of the transcript so that long transcripts do not appear more highly expressed than short transcripts, and by the number of total reads per sample to produce Reads Per Kilobase of transcript, per Million mapped (RPKM) counts. To parallel the prior tSMS data analysis plan, differentially expressed genes (DEGs) were identified by a 3-fold filter, based on absolute expression above 0.1 RPKM, a fold-change >1.5, and an uncorrected *p*-value <0.05.

For a second analytical approach that is common for the Illumina platform, the raw reads were filtered by trimmomatic [24] and then aligned to the HG38 genome using STAR aligner (version 2.5.2b) [25]. Using quantMode Gene Counts option, STAR counted the number of reads per gene while mapping. Differentially expressed genes (DEGs) were identified by using raw read counts compared between groups with DESeq2. Absolute expression levels are reported as transcripts per million (TPM). To compare the Illumina results to the SeqLL results in the overlapping 82 patients, the SeqLL results were realigned and analyzed by this method and compared without any minimal expression threshold.

#### 2.4.3 Cell type analysis

The blood-borne cell types affected by CAD were examined by using precurated lists of transcripts with preferential expression in specific cell types, as determined by RNAseq of sorted cell populations, and single cell RNAseq, as a part of the Human Protein Atlas Project [26]. The cell-type enriched transcripts, typically 10 per cell type, were used to determine an average expression level per patient, which was then averaged across patients in the LOW (n = 38), MID (n = 19), and CAD (n = 25) groups of the Discovery cohort.

#### 2.4.4 Network analysis

The WGCNA R package [27] was used to analyze the data of 28,278 transcripts measured in the combined Discovery and Validation Cohorts (N = 177). First, the transcripts with predominantly zero expression level were removed, leaving 23,675 transcripts. Based on their correlated changes across patients, transcripts were self-organized into 98 modules, each called by a unique color name. Finally, the pairwise associations between the transcript modules and sixteen clinical traits of interest were quantified. Particularly, both “gene significance” of each gene within the module to the clinical trait as well as correlation between the gene expression profile and module eigengene, which is the first principal component of a given module, were measured.

## 3 Results

### 3.1 Patient characteristics

An initial 85 patients were recruited from an ongoing cohort examining the relationship between DNA variations and CAD. A total of 82 patients had acceptable RNAseq data for further analysis, and were sequenced on both the SeqLL and Illumina platforms. A second group of 95 patients was analyzed only on the Illumina platform. The clinical and demographic analysis of the overall 177 patient cohort, summarized in Table 1, indicates that the patients with CAD were more likely to be male and older, but only the association with age was statistically significant (chi-square, p = 0.001). This suburban Virginia cohort had a lower minority composition (20 %) compared to our prior published cohort (∼50 %) at GWU in Washington, DC. As expected, CAD patients had significantly higher systolic blood pressure (BP), and higher rates of dyslipidemia, hypertension, diabetes, and smoking, but only the hypertension incidence was statistically significant (chi-square, p = 0.003) in this cohort. CAD patients trended toward lower rates of never smoking than LOW CAD patients. As expected, CAD patients were more likely to be taking daily aspirin.

### 3.2 Analytical parameters

The yield of RNA per 3 ml of blood (3.2 μg), the RNA quality (RIN 7.3, where 10 is maximum), the number of total reads (25 M per patient), filtered reads (18 M), and unique mapped reads per patient (14 M), and the percent mapped reads (81 %) did not differ between groups (Table 1). Thus, there were no detectable differences in the RNA or the RNA sequencing that would complicate the identification or interpretation of DEGs.

### 3.3 Comparison of single-molecule, amplification-free SeqLL RNAseq to Illumina RNAseq

#### 3.3.1 RPKM triple filter

**Fig. 2:**
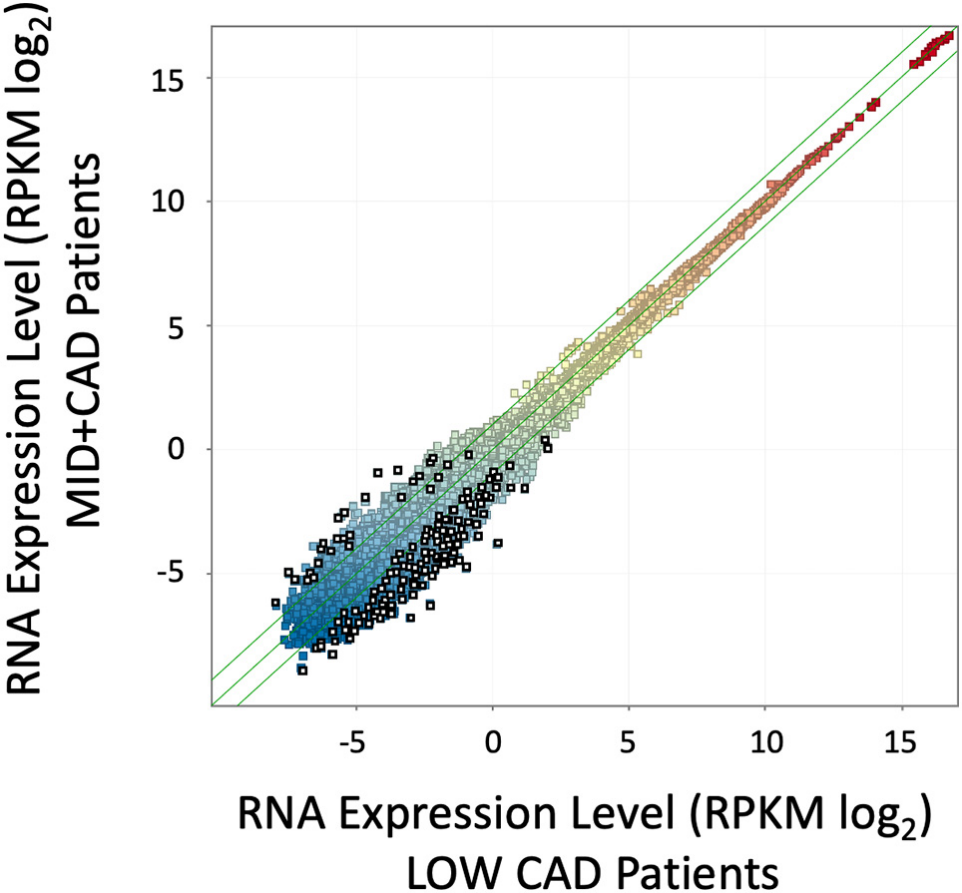
Scatterplot of RNA expression in LOW vs MID + CAD Patients. Patients presenting for elective ICA were divided into LOW CAD (<20 % stenosis, n = 38) and MID + CAD (>20 % stenosis, n = 44). Preserved whole blood RNA was analyzed by RNAseq on the Illumina Total RNA amplified platform, aligned, counted, and averaged within groups to calculate the average expression per transcript (RPKM, colored squares) in the LOW CAD group (X axis) versus the MID + CAD group (Y axis, both log_2_ scale). Differentially expressed gene (DEG) transcripts were identified by a triple filter of a minimum RPKM, >1.5-fold change, and *p* < 0.05 uncorrected for multiple testing (open black squares).

Our prior work employed amplification-free RNAseq to discover diagnostic biomarkers and relevant pathways in CAD, using a discovery cohort from GW Hospital, and a validation cohort from INOVA [18]. The validation cohort from that study (82 patients) had sufficient quantities of RNA to also be analyzed on the Illumina Total RNAseq platform using a NextSeq 500, which is reported here. Due to differences in read length between tSMS and Illumina, slightly different alignment parameters are required, but otherwise the analytical pipeline was identical. The tSMS analysis employed only an RPKM normalization with a triple filter to identify DEGs. Employing a similar approach in the Illumina data, a pattern of DEG transcripts was observed that was strikingly similar to prior tSMS patterns (Fig. 2). Consistent between the platforms, Illumina DEGs were of relatively low absolute expression level (<5 RPKM), and heavily biased (93.6 %) toward lower expression in the CAD patients, compared to 98.5 % of transcripts decreased in the published tSMS analysis [18]. Thus, the Illumina RNAseq platform identifies a pattern of changes similar to tSMS RNAseq, whereby a cluster of transcripts is found at lower levels in CAD patients.

#### 3.3.2 Comparing unamplified RNAseq to amplified RNAseq via DESeq2

To compare the two RNAseq platforms, STAR alignment and DESeq2 was employed on both datasets from the same samples, without any minimum expression threshold. DESeq2 takes a different approach to error modeling compared to RPKM/*t*-test comparisons. DESeq2 employs shrinkage estimations for dispersions and fold-changes to identify DEGs [28]. In prior comparisons of 8 analytical approaches of RNAseq data, including DESeq, the different statistical methods identified substantially different DEGs even within the same dataset of identical twins discordant for ADHD [29]. The results indicate that, overall, the quantitations in the control subjects between the platforms for the 28 K transcripts analyzed were strongly correlated (Pearson *r* = 0.938) (see Supplementary Data 2). In general, the average Illumina transcript levels (216.7 TPM) were higher than SeqLL tSMS levels (60.0 TPM), and this was especially true at the lower absolute transcript levels. This could be explained by the overall higher number of reads, and/or multiple PCR steps in the library preparation for Illumina that would increase the abundance of rarer transcripts.

**Fig. 3:**
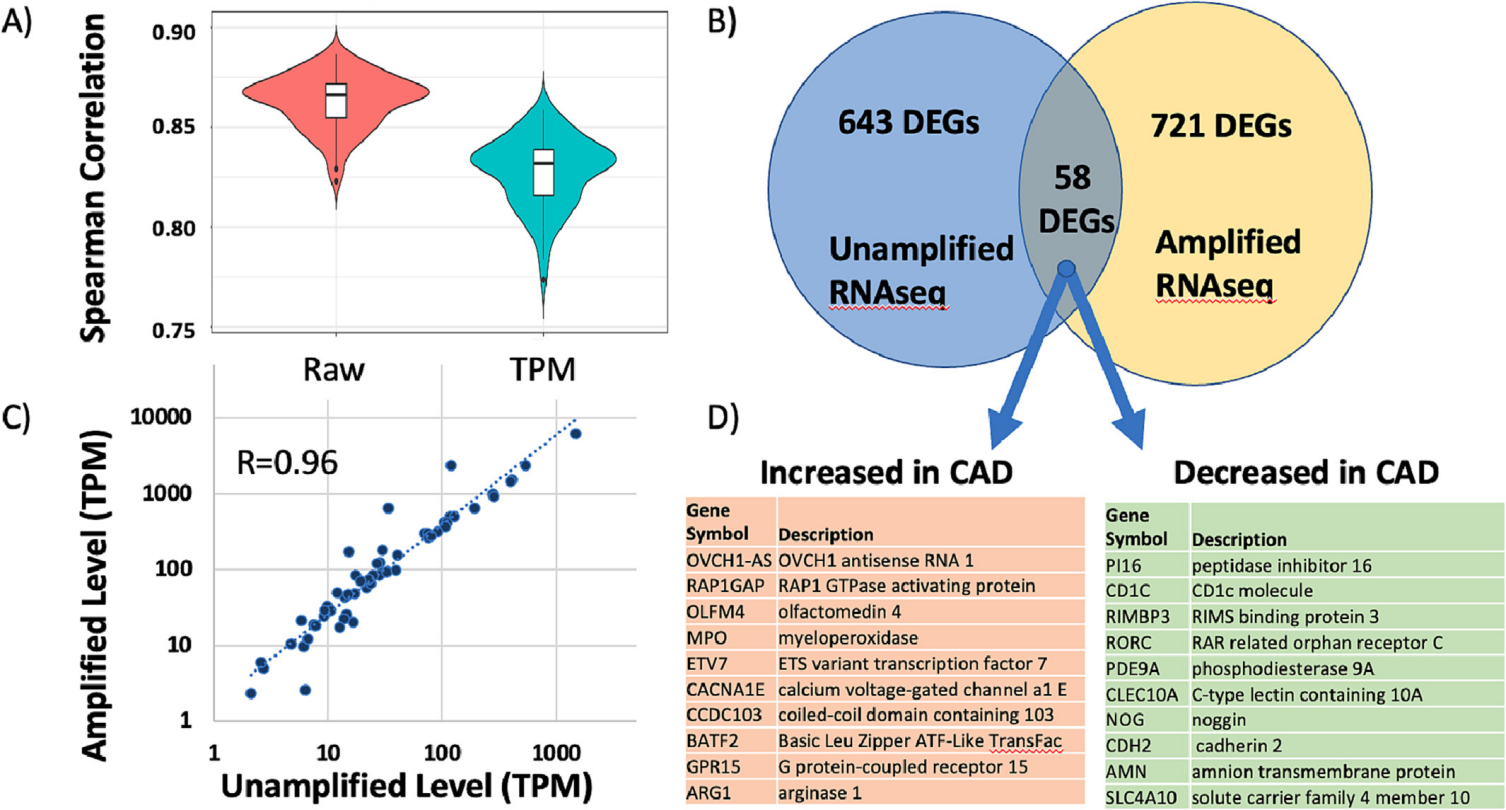
Comparison of CAD patients analyzed by SeqLL and Illumina RNAseq platforms. The patients in the Discovery cohort (n = 82) were analyzed on both SeqLL and Illumina platforms. The overall average correlation of transcript levels was 0.87 (Spearman R) for the raw transcript counts and 0.83 for TPM normalized counts, with the distributions shown by box and whiskers (Panel A). The DEGs identified by each platform had 58 common transcripts, with 57 changing in the same direction (∼9 % overlap, Panel B). Those 57 transcripts showed highly correlated quantification on the 2 platforms (Pearson r = 0.96, Panel C), and the 10 most increased and decreased transcripts are shown in Panel D.

When the expression patterns of the two platforms are examined on a patient-by-patient basis for each transcript, as opposed to across transcripts as above, the quantitative ability of the platforms decreases somewhat, but remains well-correlated, yielding Spearman correlations = 0.87 for raw read counts and r = 0.83 for TPM normalized data, as shown in Fig. 3.

Given the overall good agreement in transcript quantitation between the two platforms, a second question is whether the two platforms, SeqLL vs Illumina, identify similar DEGs. Using DESeq2 comparisons in each dataset, the transcripts were filtered to an uncorrected *p*-value of <0.05, leaving 717 DEG transcripts in the SeqLL and 788 in Illumina. However, only 61 transcripts were present in both DEG lists from the 2 platforms, with 57 of those changing in the same direction (Fig. 3). While this 8.5 % overlap is very unlikely to be due to chance occurrence (p < 6.4 × 10^−15^), it would be reasonable to expect higher concordance between the 2 platforms analyzing exactly the same RNA samples. Nonetheless, examining the correlation of absolute RNA levels (TPM) for those 58 common DEGs shows very strong quantitative agreement between platforms with R = 0.96 (Fig. 3C). Of those 58 DEG transcripts, the 10 most increased and decreased transcripts identified by both platforms is shown in Fig. 3. These shared SeqLL and Illumina DEGs echo changes seen below in the more extensive analysis of 177 patients on the Illumina platform.

### 3.4 Classification accuracy of the RNA biomarkers

In prior studies, our lab employed true single molecule sequencing (tSMS) on the SeqLL platform to develop RNA biomarkers in blood for CAD [18]. In those studies, the 7 major traditional risk factors were only slightly better than chance at predicting CAD, with an area under the curve (AUC) of 0.636 where 0.5 is random chance, or roughly 54 % accuracy. This diagnostic uncertainty is confirmed in the present study by the relatively weak associations of risk factors like dyslipidemia, hypertension, diabetes, and family history, as shown in Table 1. By comparison, a set of 7 transcripts in that prior dataset was 84 % accurate at diagnosing CAD.

In the present study, the top 10 increased and top 10 decreased DEGs by Illumina RNAseq in the Discovery Cohort were used to build a simple predictive model for CAD, but surprisingly, the model was only about 25 % sensitive at detecting CAD, in effect, less accurate than random chance. Thus, while the DEGs identified by Illumina RNAseq are generally associated with CAD, they are poorly predictive, likely due to either: 1) the relatively low absolute expression level of the DEGs (0.01–5 TPM), 2) the possible presence of multiple subgroups of CAD transcripts that complicate diagnosis, or 3) relatively poor reproducibility in quantifying the transcripts, which inserts unacceptable noise into the predictive algorithm. Restricting the DEGs to higher TPM levels did not drastically improve the predictive ability. The possibility of subgroups or quantitative errors in the amplified Illumina RNAseq are evaluated by further analyses below.

### 3.5 Ontology/pathway analysis of RNA biomarkers

The DEG transcripts identified by the Illumina amplified RNAseq were subjected to relatively unbiased analysis by comparison to pre-curated gene ontologies and pathways. To provide the best overall power, the Discovery and Validation cohorts were merged to create a combined cohort of 177 patients, that was analyzed by DESeq2, which uses a negative binomial distribution and Wald test for each transcript to identify DEGs. Applying the DESeq2 analysis to this merged cohort identified 933 DEG transcripts, which was filtered to 102 transcripts with an uncorrected p-value of <0.05 and an absolute fold change >1.5-fold (61 increased, 41 decreased, Supplementary Data 3). Because these transcripts do not show a normal distribution, we further tested them with a non-parametric Mann-Whitney test and found that 50 % were significantly different at p < 0.05 uncorrected, and 25 % with multiple testing correction (Supp. Data 3). These 102 DEGs from the combined 172 patient cohort were submitted for gene ontology analysis, via NIH DAVID [30], and by Ingenuity Pathway Analysis (IPA). On the basis of this ontology/pathway analysis, as well as extensive manual curation, some of the most interesting DEGs modulated in CAD are shown in Table 2.

There was an obvious enrichment in the DEGs for cardiovascular disease in general (11 transcripts p < 0.03), and especially CAD related transcripts. IPA found that at least 6 of the transcripts have known associations with CAD (KCNG2, FN1, KCNJ11, TUBB, BMPR1B, KCNA5; p < 0.002), with several others identified by manual curation: FMOD, PIGR, GLDN, FIGN, and RRC32, for example.

**Table 2:** Annotation of select differentially expressed genes in CAD.

Gene	Description	Expression	Fold	Pathway	Cell type
**Symbol**	**Increased Transcript Levels**	**Level (TPM)**	**Change**		
FMOD	Fibromodulin	0.46	2.83	TGF	T cells
TEAD1	TEA domain transcription factor 1	0.76	2.09	Hippo	T cell, mem CD4+
PIGR	Polymeric immunoglobulin receptor	1.27	2.08	Immune	Epithelial
TMEM98	Transmembrane Protein 98	1.01	2.07	Wnt/catenin	Th1 differentiation
DCDC2B	Doublecortin domain containing 2B	0.47	1.96	Cilia	Auditory hair cells
GLDN	Gliomedin	0.72	1.98	Nodal	Neural
MIR548	MIR548A1 host gene	0.42	1.95	TLR	Immune
MSLN	Mesothelin	0.91	1.91	Stereocilin	Treg
FLOT1	Flotillin 1	5.98	1.90	Immune	T cell, CD8+
RAP1GAP	RAP1 GTPase activating protein	99.46	1.77	GTP	Multiple
FN1	Fibronectin 1	10.59	1.73	Adhesion	T cells
FAP	Fibroblast activation protein alpha	1.01	1.71	Inflammation	Fibroblast
BTN1A1	Butyrophilin subfamily 1 member A1	0.70	1.71	T cell signals	T cells
TUBB	Tubulin beta class I	11.71	1.67	CAD	B cells
GIMAP5	GTPase, IMAP family member 5	0.66	1.63	GTP	T cell, CD4+
FIGN	Fidgetin, microtubule severing factor	1.74	1.60	Microtubule	Neural
ETV7	ETS variant transcription factor 7	32.04	1.60	Apoptosis	T cell, CD8+
LRRC32	Leucine rich repeat containing 32	2.06	1.50	Immune	Treg

**Symbol**	**Decreased Transcript Levels**	**Expression**	**Fold**	**Pathway**	**Cell type**
ECHDC3	Enoyl-CoA hydratase domain containing 3	30.01	1.52	Fatty acid	Lymphocyte
STRC	Stereocilin	0.71	1.54	Ciliary	Auditory hair cells
LRRC7	Leucine rich repeat containing 7	9.04	1.55	Polarity	Epithelial, synaptic
NRCAM	Neuronal cell adhesion molecule	7.90	1.55	WNT GLDN	T cell, CD4+
EIF2S3B	Euk. translation initiation factor 2 S3B	8.68	1.67	GTP	Multiple
SIAH3	Siah E3 ubiquitin protein ligase 3	0.85	1.68	Autophagy	Multiple
BHLHA15	Basic helix-loop-helix family member a15	0.95	1.69	ER stress	Plasma cell
SFRP5	Secreted frizzled related protein 5	0.75	1.71	Wnt, obesity	White adipose
SCGB3A1	secretoglobin family 3A member 1	1.19	1.73	Fusion	Myoblast
TXNDC5	Thioredoxin domain containing 5	1.24	1.74	Nitric Oxide	Endothelial
SHISA2	Shisa family member 2	1.03	1.95	Wnt signaling	Multiple
NEBL	Nebulette	41.83	2.38	Cytoskeletal	Cardiac muscle

A more unexpected enrichment was that both DAVID and IPA detected enrichment of synaptic-like transcripts that would normally be associated with neural function, and thus seem unusual in the context of whole blood. There was a noticeable enrichment for functions related to cell-to-cell signaling (22 transcripts, p < 0.00005), which is surprising because blood is typically envisioned as ‘free floating’ cells with little direct cell-cell contact. More surprising, in the cell-to-cell signaling category, the strongest enrichment is for ‘synaptic transmission’ (6 transcripts: ADCY1, APBA2, HTR1B, LYPD1, NRCAM, OMP; p < 0.003).

#### 3.5.1 Cell type-specific RNA markers in relation to CAD level

To explore the hypothesis that the RNA signature is related to particular blood cell types, published single-cell RNAseq (scRNAseq) and sorted cell analysis of human blood cell types [26] was cross-referenced to the current RNAseq transcriptome, and used to build a composite index of ∼10–20 mRNAs relatively unique to each subtype. The particular cell-type enriched transcripts employed, with their enrichment scores and levels in each group, and in additional cell types, are found in Supplementary Data 4. Notably, in our prior studies on the tSMS platform, a composite index of RNA expression levels showed a trend toward lower expression of T cell markers in patients with CAD [18].

**Fig. 4:**
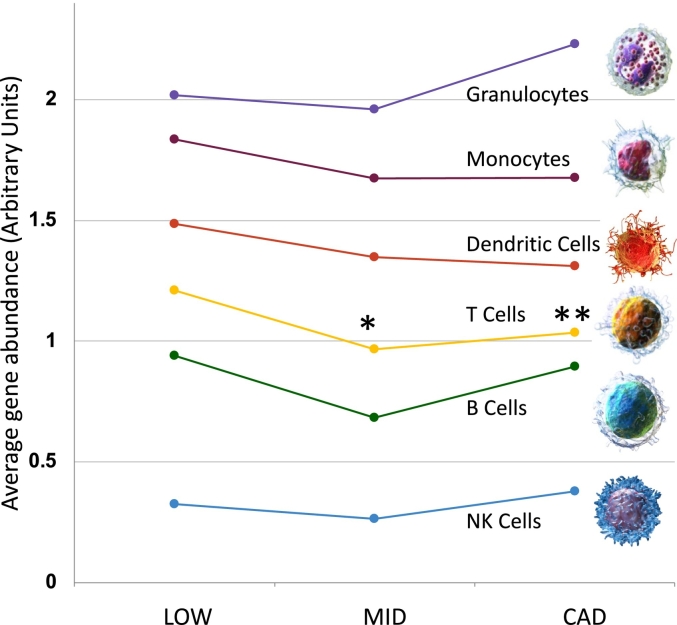
Cell type-specific RNA transcript expression in CAD groups. A pre-curated set of transcripts with relative enrichment in particular blood cell types was derived from single cell RNAseq data from the Human Blood Atlas portal [26]. RNAseq levels were computed for each cell-type set (∼10–20 transcripts per cell type) in LOW (n = 38), MID (n = 19), and CAD (n = 25) groups of the Discovery cohort (n = 82). Asterisks indicate average transcript levels compared by a paired *t-*test between LOW and MID, or LOW and CAD groups has a p-value <0.05 (*) or 0.01 (**) uncorrected for multiple testing. More detailed subsets of cells (e.g. Treg, or B memory) can be found in Supplementary Data 4. Cell type-specific RNA transcript expression in CAD groups. A pre-curated set of transcripts with relative enrichment in particular blood cell types was derived from single cell RNAseq data from the Human Blood Atlas portal [26]. RNAseq levels were computed for each cell-type set (∼10–20 transcripts per cell type) in LOW (n = 38), MID (n = 19), and CAD (n = 25) groups of the Discovery cohort (n = 82). Asterisks indicate average transcript levels compared by a paired *t-*test between LOW and MID, or LOW and CAD groups has a p-value <0.05 (*) or 0.01 (**) uncorrected for multiple testing. More detailed subsets of cells (e.g. Treg, or B memory) can be found in Supplementary Data 4.

In the present Illumina RNAseq, the relative reduction in T cell transcripts in MID (p < 0.05) and CAD (p < 0.1) groups is reproduced, despite a different cohort and RNAseq platform (Fig. 4). However, in this Illumina dataset, there are comparable differences, although not statistically significant, in B cells, dendritic cells and monocytes that were not observed with the tSMS analysis. In contrast, granulocyte and NK cell transcripts were elevated, but not significantly, in the CAD group. While one might expect a stepwise reduction in the cell-enriched transcripts from LOW to MID to CAD, in fact, both the prior and current studies observed the greatest reductions in the MID group, with either no further reduction, or even recovery in the more severe CAD group. For reference, a group of 50 random transcripts did not show a pattern comparable to any of these blood cell types. The Blood Atlas datasets contains more refined subsets of cells, such as Treg, naïve T cells, gamma delta T cells, etc., but there were not obvious subsets affected by CAD (see Supplementary Data 4 for detailed breakdown).

#### 3.5.2 Transcription factor analysis of DEGs by DESeq2

Analysis of shared transcription factors by the 61 upregulated transcripts, using CHEA3 software [31], suggests the top 5 shared transcription factors were HEYL (8 overlapping genes), MEOX (6), FOXC2 (6), FOXS1 (6) and AEBP1 (5). By submitting the 41 down-regulated genes for analysis of common transcriptional regulators, the most likely factors were CSRNP3, DLX1, DACH2, FOXG1, and importantly SMYD3, which our prior publication identified as a DEG in CAD by tSMS [18]. SMYD3 is intimately involved in the FOXP3 transcriptional program, and downregulated itself in CAD patients when analyzed by tSMS.

DESeq2 analysis of both the Illumina and tSMS data identified Ets variant 7 (ETV7) as differentially expressed in the CAD patients (Fig. 3D), and our prior work likewise identified Ets1 as varying in relation to the degree of CAD [18]. Likewise, the earliest microarray analysis of whole blood RNA changes in CAD identified SPIB, a member of the Ets family, as increased in CAD [11]. Published studies indicate Ets transcription factors are intimately involved in the differentiation of T cells along the Treg pathway [32]. An extensive analysis of regulatory elements in the immune response to cancer types revealed ETV7 as strong regulator of the T cell receptor signaling pathway [33], and thus, ETV7 is a diagnostic marker of CD8+ T cell infiltration in melanoma [34] and urothelial cancer [35]. GWAS studies found that Ets1 variants are associated with autoimmune SLE [36]. However, multiple studies examining ETV7 over- or under-expression suggested ETV7 is a repressor of the interferon-response transcripts [37], [38], a pattern that we cannot confirm in the present studies (not shown). Thus, the elevated ETV7 is likely a marker of accumulation of T cells in the CD8+ lineage, possibly related to reduced differentiation into the Treg pathway. Considering the consistent appearance of the ETS members in human CAD analysis, the involvement of Ets family members warrants further consideration in the immune components of CAD.

#### 3.5.3 Weighted gene co-expression analysis of the combined cohort

**Fig. 5:**
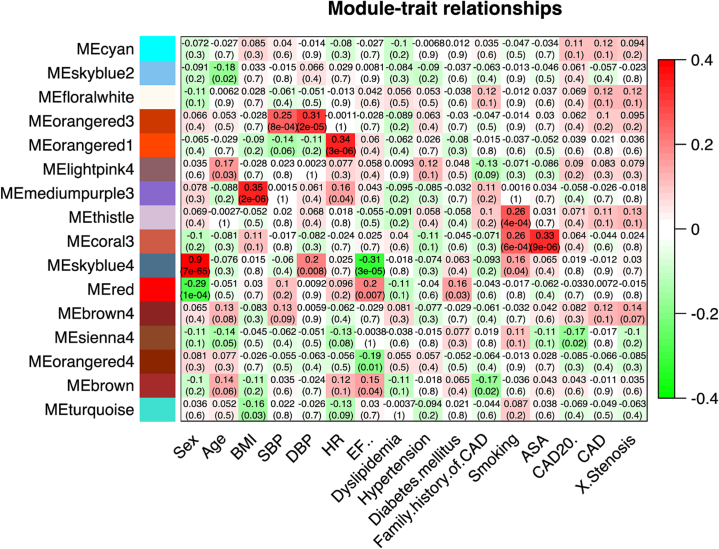
Weighted Gene Co-Expression Analysis (WGCNA) of CAD-related transcripts. The raw expression data of 28 K transcripts from the combined cohort of 177 patients was analyzed for covariant modules of transcripts across patients without regarding to any clinical grouping. A total of 98 covariant modules were identified, and then their association with 16 clinical traits was analyzed to yield a module-by-trait association, which was then filtered to show the most strongly associated modules (Y axis) with each of clinical traits (X axis) color-coded by a positive (red) or negative (green) correlation, with the p-value of the association in parentheses. (For interpretation of the references to color in this figure legend, the reader is referred to the web version of this article.)

To understand any deeper patterns in the expression data, and to identify potential subgroups of transcripts that might carry some collective classification value, the 177-patient cohort analyzed for 28 K transcripts was subject to weighted gene co-expression network analysis (WGCNA). In short, WGCNA identifies clusters of covariant transcripts and then determines their relationship to the defined traits of interest [27]. The network analysis conducted correlational analysis to identify 98 distinct ‘modules’ of transcripts that vary in a coordinate pattern across the patients, without knowledge of any clinical or experimental traits. Those 98 expression modules, which contain from 1 to 9523 transcripts per module, were then associated with 16 selected clinical traits to find gene sets that track the clinical trait, as shown in Fig. 5. For example, the arbitrarily named MEskyblue4 module contains 30 intercorrelated transcripts that are highly associated with the sex trait (0.9, p = 7 × 10^−65^), and all the transcripts are either on the X or Y chromosome. Several transcript modules are found to correlate with clinical traits at lesser, but significant correlations such as BMI with MEmediumpurple3 (0.35, p = 2 × 10^−6^); systolic and diastolic blood pressure with MEorangered3 (0.25, p = 8 × 10^−4^ and 0.31, p = 2 × 10^−5^, respectively); heart rate (0.34, p = 4 × 10^−4^) and smoking (0.26, p = 6 × 10^−4^) with MEthistle and MEcoral3; and ASA consumption with MEcoral3 (0.33, p = 9 × 10^−6^).

The CAD trait, at 20 % stenosis, is significantly associated with only one module (MEsienna4, −0.17, p = 0.02), and it contains only 3 transcripts: APBA2, CIART, and LINC00479. APBA2, the amyloid ß precursor protein binding A2 (aka X11ß, MINT2), is principally recognized for its role in synaptic vesicle exocytosis and its ability to stabilize amyloid precursor protein (APP), and is considered part of the genetic signature of Alzheimer's disease in Down syndrome [39]. By DESeq2 analysis, APBA2 transcript is expressed at a low level in blood (1.4 TPM) and is decreased 1.8-fold (p = 0.009) in CAD patients. Of relevance to CAD, APBA2 facilitates stress-induced phosphorylation of APP [40], and could have some function at the immune synapse. Thus, persistent immune stress on T cells from CAD patients may affect the immune synapse.

CIART is a circadian related transcript that has little relation to CAD on its own, and is not differentially expressed by DESeq2, while LINC0047 is slightly lower in CAD patients (12 %) but is a non-coding RNA with no known association to CAD-relevant parameters. Thus, this module is mostly powered by APBA2 with 2 correlated transcripts of unknown relevance to CAD.

Three other correlated modules are larger gene sets (81–143 transcripts), but are associated with CAD at lower strength. The MEcyan module (0.11) in Fig. 5, appears to track RBC, because it contains several hemoglobin subunit RNAs (HBD, HBG2, HBM). The similarly associated MEdarkorange module (−0.11) is composed of 81 transcripts containing 24 long intergenic non-coding transcripts (LINCs) or microRNAs. Of the annotated transcripts, their functions are diverse, but one, in particular, suggests the module is immune related: CCL8 is a CCR5 ligand that is an inflammatory chemokine with activity on T cells [41].

Another CAD correlated module, MEturquoise (−0.069), contains 9523 transcripts that appear to be a broad T lymphocyte cluster because it contains a variety of transcripts that we and others have associated with T/Treg cells, such as DGKA, SMYD3, and TRIM28 [18], as well as consensus T cell markers such as CD3, CD4, and CD8. The MEfloralwhite module (0.069) gains some strengthened association using a more stringent CAD trait of 70 % stenosis (CAD70, 0.12) or the continuous variable of %stenosis (0.12). The MEfloralwhite module is composed of 60 transcripts of which 26 are either long non-coding RNA or microRNA, with the remaining transcripts apparently linked to signaling.

#### 3.5.4 Potential involvement of cilia/immune synapse transcripts

There appeared to be a disproportionate number of transcripts related to ciliary and synaptic functions in both the prior single-molecule RNAseq as well as in the present amplified RNAseq. The presence of these transcripts in blood is notable because, upon closer examination, it became evident that they were likely related to the function of the immune synapse. In particular, transcripts like stereocilin (STRC, down 1.5-fold in CAD) encode proteins that are known to be involved in the stereocilia of the outer hair cells in the inner ear, and their mutation can lead to congenital hearing loss [42]. Interestingly, STRC is known to interact with mesothelin (MSLN, increased 1.9-fold in CAD), and both are superhelical lectins with ARM-type repeats that bind carbohydrate groups in extracellular glycoproteins [43]. Another cilia-related transcript is doublecortin-domain containing 2B (DCDC2B, increased 1.9-fold in CAD) which interacts with tubulin 2B (TUBB, increased 1.7-fold in CAD) to affect the hair cell kinocilia, and its mutation likewise leads to recessive deafness [44].

### 3.6 Comparison of Illumina biomarkers to IonTorrent biomarker panels

A recently published dataset using 52 patients undergoing coronary computed tomography angiography (CTA) compared whole blood RNA, similar to the current studies, but subjected to globin depletion and polyA enrichment prior to being sequenced by the IonTorrent RNAseq methodology [45]. At a high level, the patterns are similar because Andreini et al. also observed the significant reductions in T cell related transcripts, with a lesser effect on B cell transcripts, and increased expression of neutrophil transcripts [45]. However, at the level of the specific transcripts, comparing their 138 top DEGs to the current 100 top DEGs, there were no identical matches. Some similar transcripts were observed: ETV3 vs ETV7, LENG1 vs LENG8, MARCHF9 vs MARCHF10. However, it is difficult to know the odds of similar transcripts being identified by chance.

### 3.7 Reproducibility of RNAseq quantitation on the Illumina platform

To determine the degree to which the variance between cohorts, and RNAseq platforms, could at least be partially explained by variance in the quantitation of the expression levels, 9 patients were sequenced twice on sequential runs using the identical Illumina Total RNA protocol, as used above. While the overall correlation of gene expression across transcripts is quite good (r = 0.98), when the correlations between runs are measured within the individual transcripts across patients, there is a full range of correlations observed, with the median correlation for a given transcript was only 0.621 (Pearson r) or 0.603 (Spearman rho). Shown as the difference between the two runs as a percent of the average absolute value, the range of errors is shown in Supplementary Data 5, with positive and negative errors of up to 200 % each. The aligned reads for these samples did not differ significantly between runs (8.9 vs 9.7 M aligned, paired *t*-test p = 0.29) and were poorly correlated (Pearson r = −0.49).

The run-to-run variance was generally higher as the absolute abundance of the transcript decreased, but as much as 100 % deviation is apparent even at the relatively high expression of 100 RPKM. Examining the 1000 most divergent transcripts, defined as a Pearson r < −0.28 between runs, indicates that 80 % are <1 RPKM in abundance, and 80 % of those remaining are relatively small transcripts, typically miRNAs. Further restricting the analysis to transcripts >1500 nt yields 38 transcripts that mimics the size of typical transcripts (average = 3164 nt). There is a known sensitivity of Illumina RNAseq to the size and GC content of the transcripts [46], however these exemplars had the expected GC content (mean = 47 %, range 40–60 %).

Thus, while RNAseq is very powerful in relative quantitation within a given sample over a very broad range of absolute RNA abundance, its ability to quantitate any particular transcript reproducibly, even in the same sample, is quite unpredictable. This level of performance is adequate, if not powerful, for the discovery of general patterns of differential expression, but it is cautionary for utilizing RNAseq in a clinical diagnostic manner, where the reproducible quantitation of individual transcripts is essential.

## 4 Discussion

### 4.1 The immune-atherosclerosis theory

A rapidly growing literature is documenting the intimate and likely causative connection between the immune system and atherosclerosis. The earliest pathological examinations of atherosclerotic plaques observed the presence of monocytes/macrophages, neutrophils, lymphocytes, and platelets [47], [48], [49]. Recent single-cell RNAseq (scRNAseq) of human carotid plaques has identified at least 14 subtypes of cells, including several T cell subsets [50]. There are highly reproducible changes in the Treg/Teff ratio in patients with CAD [19], [20], [51], [52], that are consistent with the detected changes in mRNA expression in the present studies. The cytokine responsiveness of T cell subsets is a better predictor of CAD than C-reactive protein (CRP) levels in patients [53]. A variety of lines of evidence suggest that Treg dysregulation mechanistically contributes to the development of CAD [54]. Experimental manipulation of Treg levels in mouse models of atherosclerosis suggests a causal relationship between Treg deficiency and plaque progression [55]. Mechanistically, recent studies indicate that Tregs license the pro-resolving abilities of plaque-resident macrophages in order to facilitate plaque regression [56]. Interestingly, a plasma proteomics study, profiling of hundreds of circulating proteins, also points to a role for T cell dysfunction in congestive heart failure [57].

### 4.2 Atherosclerosis and autoimmunity

Clinically, the relationship between Treg dysfunction and atherosclerosis is prominently observed through the well-known incidence of atherosclerosis in various autoimmune diseases, most notably in the case of systemic lupus erythematosus (SLE) [58]. Deficient Treg activity is one element of SLE [59], and likely contributes to SLE-associated atherosclerosis [60]. Likewise, psoriasis and psoriatic arthritis, which are associated with Treg imbalances, have well-established associations with atherosclerosis [61], [62], [63]. Furthermore, sequencing of circulating and plaque-resident T cell receptors, combined with flow cytometry and RNAseq, identifies clonal expansion of effector T cells in carotid plaques, and implicates a psoriasis-like autoimmune component to atherosclerosis [64].

The immune-CAD connection is seen quite clearly by an apparently causal relationship in immune-mediated transplant arteriosclerosis [65]. Conversely, rapamycin, an immunosuppressant, is known to increase Treg numbers and function at clinically relevant levels, and it is used on drug coated stents to control coronary stenosis [66]. The compelling connection between autoimmunity and atherosclerosis has suggested that a Treg-oriented immunomodulatory approach may offer therapeutic potential for atherosclerosis [67], [68]. It is already known that in addition to lowering LDL cholesterol, statins, which are widely prescribed to reduce CAD risk, can induce FoxP3+ Treg cells, via modulation of TGF-ß signaling [69], [70].

### 4.3 The RNA signature of CAD

The present studies add several novel dimensions to our current understanding of CAD. The current studies employed a completely different RNAseq platform, and yet identified a pattern of RNA changes similar to our prior RNAseq studies. Both analyses point to changes mainly in the T cell subset, and are consistent with a Treg-type of dysfunction. Further, the new transcripts identified by both the single-molecule and amplified Illumina platform further narrows and increases the confidence in those targets. The present studies are by far the largest and most in depth RNAseq analysis of CAD to date.

**Fig. 6:**
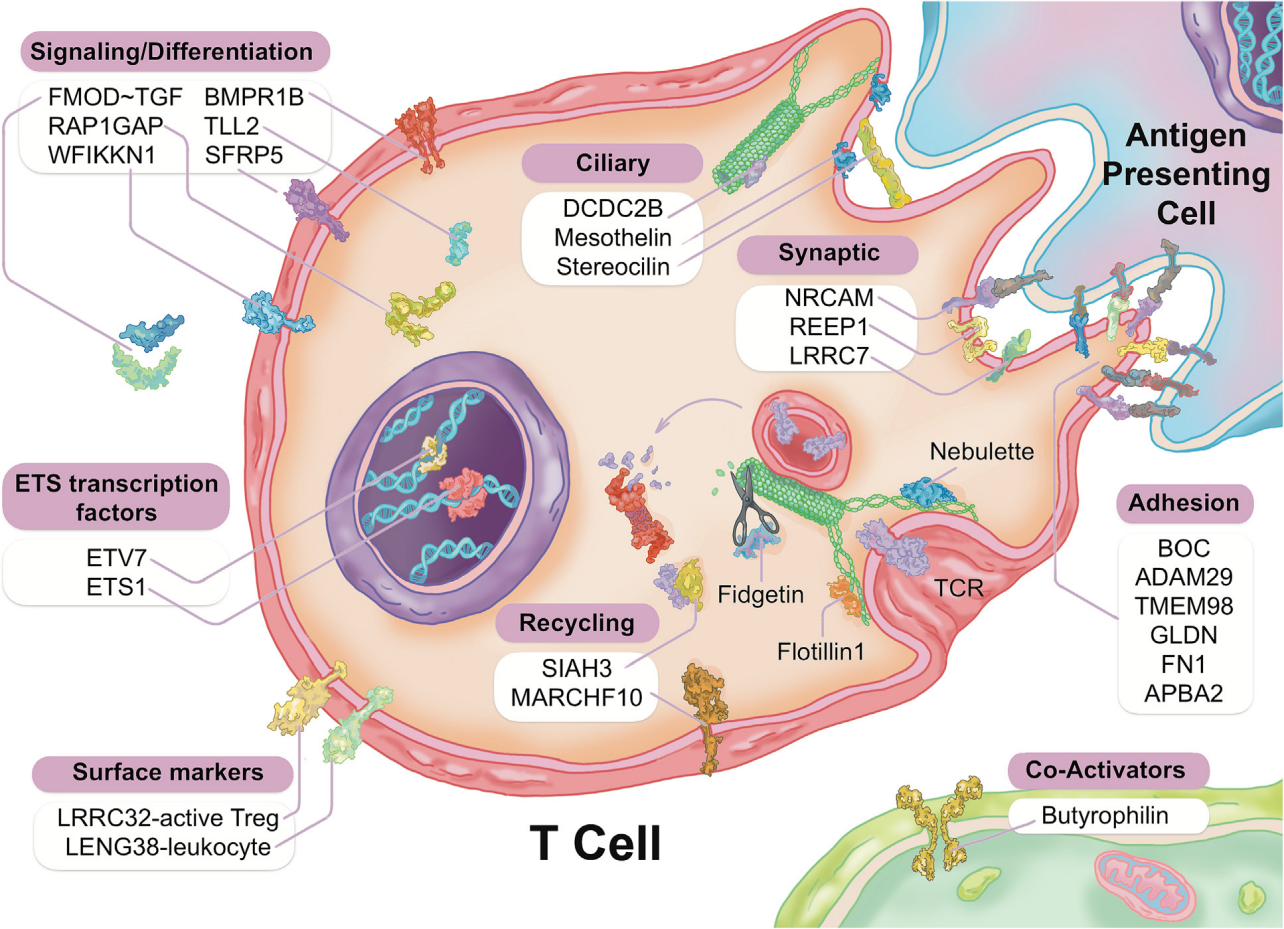
Schematic representation of Treg-related TRACs identified by RNAseq. The differentially expressed genes (DEGs) identified by RNAseq were curated by automated and manual analysis to identify the molecular pathways involved. The resulting pattern points to changes in the ‘immune synapse’, which involves both endocytic pathways of T cell receptor (TCR)-containing vesicles, as well as ciliary protrusions that couple to intracellular signaling pathways.

As summarized in Fig. 6, the RNA changes are mostly related to ciliary and endocytic transcripts, which in the circulating immune system would be related to the immune synapse. The immune synapse is the contact-dependent mode of communication between T cells and B cells, on one side, and a variety of antigen-presenting and immunomodulating cells on the other side. One of the most differentially expressed genes, fibromodulin (FMOD, increased 2.8-fold in CAD), has a known connection to atherosclerosis [71], [72], cardiomyopathy [73] and ferroptosis (iron-dependent programmed cell death) [74]. Coincidentally, other groups have conducted meta-analysis, including our prior single-molecule RNAseq data, and observed ferroptosis-related subgroups of CAD patients [75]. As shown, fibromodulin is intimately involved in the endocytosis of T cell receptor (TCR) containing vesicles that are essential to TCR signaling, processing, and recycling.

Several other regulated transcripts encode for proteins related to the structure and function of the immune synapse. Nebulette, the most down-regulated transcript (2.4-fold), is an important ‘cytolinker’ that connects actin and desmin to facilitate cytoskeletal function and vesicular movement [76]. The endocytic pathway is further modulated by changes in tubulin, which is a key microtubule protein, and fidgetin, which is a tubulin-severing enzyme that is a GWAS marker for CV risk [77]. Protein recycling would be modulated by changes in the proteasomal regulator SIAH3, and the ubiquitin ligase MARCHF10. On the ciliary aspect of the immune synapse, several of the modulated transcripts are related to ciliary length and function. Steriocilin (STRC) has been studied principally in outer sensory hair cells, and mutations lead to deafness [42]. Steriocilin is a partner to mesothelin (MSLN), a related super-helical protein [43], whose transcript is also modulated in CAD. Likewise, DCDC2, a double-cortin protein, is a known modulator of ciliary length [44].

In the signaling pathways of the immune synapse, there were numerous transcripts that related directly to T cell function and the control of differentiation. Butyrophilin (BTN1A1) is a known co-regulator for T cell activation [78]. Fibromodulin is a well-known modulator of the TGF-ß signaling pathway [79], which is a primary determinant of Treg differentiation [80]. Further impact on the TGF-ß pathway is reflected in concurrent changes in the BMP receptor 1B RNA (BMPR1B), because the bone morphogenic proteins are members of the TGF-ß superfamily, and likewise impact Treg differentiation [80]. As noted, several of the transcripts (TMEM98, NRCAM, SFRP5, SHISA2) are known elements of the Wnt signaling pathway, which is major determinant of Treg differentiation [81].

### 4.4 Limitations to the current studies

Technically, it is quite surprising that these 2 advanced RNAseq platforms exhibit only about 10 % concordance at identifying transcriptome changes. However, the 2 platforms showed an overall strong quantitative correlation across transcripts, but there is considerable variability in the ability to quantify any single transcript reproducibly. This should be cautionary for the near-term clinical utility of RNAseq, and should encourage investigators to be more diligent about ensuring sufficient group sizes in RNAseq studies to overcome this technical variability.

Clinically, it is likely that the blood RNA biomarkers, once refined, would detect atherosclerotic disease in arteries other than the coronaries, but this would still have tremendous diagnostic value. A potential concern is that the transcriptome changes could be related to unidentified risk factors or drug treatments that differ between groups. However, using a variety of methods, we cannot identify a clinical covariate that would differ sufficiently to create this effect. A significant limitation is that the clinical endpoint of an invasive coronary angiography (ICA) is excellent, but still imperfect at detecting coronary disease. Probably 75 % of symptomatic patients that appear to have normal arteries by ICA are shown by CTA to have significant atherosclerosis that does not occlude the artery [82]. The RNA biomarkers should be altered in these cases even thought they would be scored as angiographically normal by ICA. Future studies will need to incorporate CTA to provide the most accurate clinical diagnosis.

### 4.5 Future directions

The present studies suggest several important directions for future investigation. Through high-throughput screening, dozens of FDA-approved compounds that stimulate Treg generation have already been identified [83]. Further refinement in the quantitation of the RNA biomarkers, especially by pre-enrichment of T cells from whole blood, could lead to blood-based diagnostics for CAD, that would be a valuable addition to the diagnostic toolkit. A long-term goal is to identify RNA biomarkers that may be predictive of CAD in asymptomatic, but ‘at risk’ individuals, especially those at intermediate risk of CAD. About 50 % of heart attacks have no overt warning signs, and 50 % of first heart attacks are fatal. Thus, an ‘early warning sign’ from blood-based RNA profiling could allow the patient to be referred for minimally invasive diagnostics, such as stress tests, CT calcium scores, or MR/CT angiography, and thus hopefully reduce the incidence of heart attacks and strokes.

### 4.6 Conclusions

Transcriptome-wide profiling of whole blood RNA on 2 distinct RNAseq platforms using a large cohort of patients with CAD identifies a pattern of changes that parallels known dysfunction of the regulatory T cell subset. Despite a strong overall correlation between the 2 RNAseq platforms, there is only about a 10 % concordance in the RNA changes that they identify. The RNA changes are consistent with T cell-related changes in the immune synapse, which may help to define the precise cellular mechanisms of atherosclerotic lesion formation and suggest optimal diagnostic and therapeutic targets. Mechanistically, the RNA targets seem centered around the ciliary and immune synapse pathways, which provides a novel starting point for interventions.

The following are the supplementary data related to this article.

Supplementary data 1 Analytical workflow for RNAseq of human blood RNA Supplementary data 1

Supplementary Data 2 Correlation of SeqLL vs Illumina baseline TPM per transcript. Supplementary Data 2

Supplementary Data 3 Illumina DEG transcripts annotated (177 patients) Supplementary Data 3

Supplementary Data 4 Cell-type specific transcripts of all subsets. Supplementary Data 4

Supplementary Data 5 Illumina run to run variation by RPKM Supplementary Data 5

Supplementary Data 6 Galaxy workflow for RNAseq analysis. Supplementary Data 6

## Ethics approval and consent to participate

All subjects gave written, informed consent under IRB Protocol #111015, approved by The George Washington University Institutional Review Board, and Protocol # 15–2168 approved by the INOVA Fairfax IRB.

## Consent for publication

Not applicable.

## Availability of data and materials

The expression-level data from the single molecule sequencing is deposited in the Gene Expression Omnibus (GEO) at the accession #GSE180083. The expression level data from the Illumina RNAseq, as both TPM and raw read counts, is available at accession #GSE221911. The sequence level data from this study will be provided to qualified investigators that can ensure compliance with appropriate IRB and HIPPAA regulations for any future data usage, by contacting the corresponding author at mcc@gwu.edu. The human genome files for alignment were obtained from UCSC at this link for HG38 (https://hgdownload.soe.ucsc.edu/goldenPath/hg38/bigZips/). The Galaxy analytical workflow is available as Supplementary Data 6.

## Funding

The authors are grateful for the financial support of the 10.13039/100007108GW Heart and Vascular Institute, True Bearing Diagnostics, Inc. and The St. Laurent Institute, without which the studies would not have been possible. The authors are also grateful for the support of Award Number UL1TR001876 from the NIH 10.13039/100006108National Center for Advancing Translational Sciences, and Core Instrument Grant for the BioRad ddPCR S10 OD021622.

## CRediT authorship contribution statement

G.S.L., R.K., I.T., and T.A.M. conceived and designed the studies. P.S., J.R., R.K., and R.M. identified and consented patients, collected clinical and laboratory data, and contributed clinical expertise on the conduct and analysis of the studies. Z.Y., I.T., D.J., D.S., and T.J., conducted RNA isolations, RNA sequencing, and alignments. D.S., D.A., T.A.M., Z.F., R.W., M.R., J.G., Z.F., A.F., K.J., and Y.L. conducted the statistical, annotation, and bioinformatic analyses. N.R. assisted in pathway analysis and graphic presentations. T.A.M. wrote the manuscript with input from all the authors.

## Declaration of competing interest

TM, TJ, and IT have an equity interest in True Bearing Diagnostics, Inc., a diagnostics company developing RNA biomarkers for various diseases, including coronary artery disease. IT, GSL3, RK, and TM are seeking patent protection for a commercial diagnostic test, without restriction for research uses. DJ is the CEO of, and equity holder in SeqLL, Inc.
