# Supplementary figures and images for: RNAseq profiling of blood from patients with coronary artery disease: Signature of a T cell imbalance
Source: J Mol Cell Cardiol Plus. 2023 Mar 25;4:100033. doi: 10.1016/j.jmccpl.2023.100033 (PMC10256136; doi:10.1016/j.jmccpl.2023.100033)

# Supplementary Data 1. Analytical workflow.

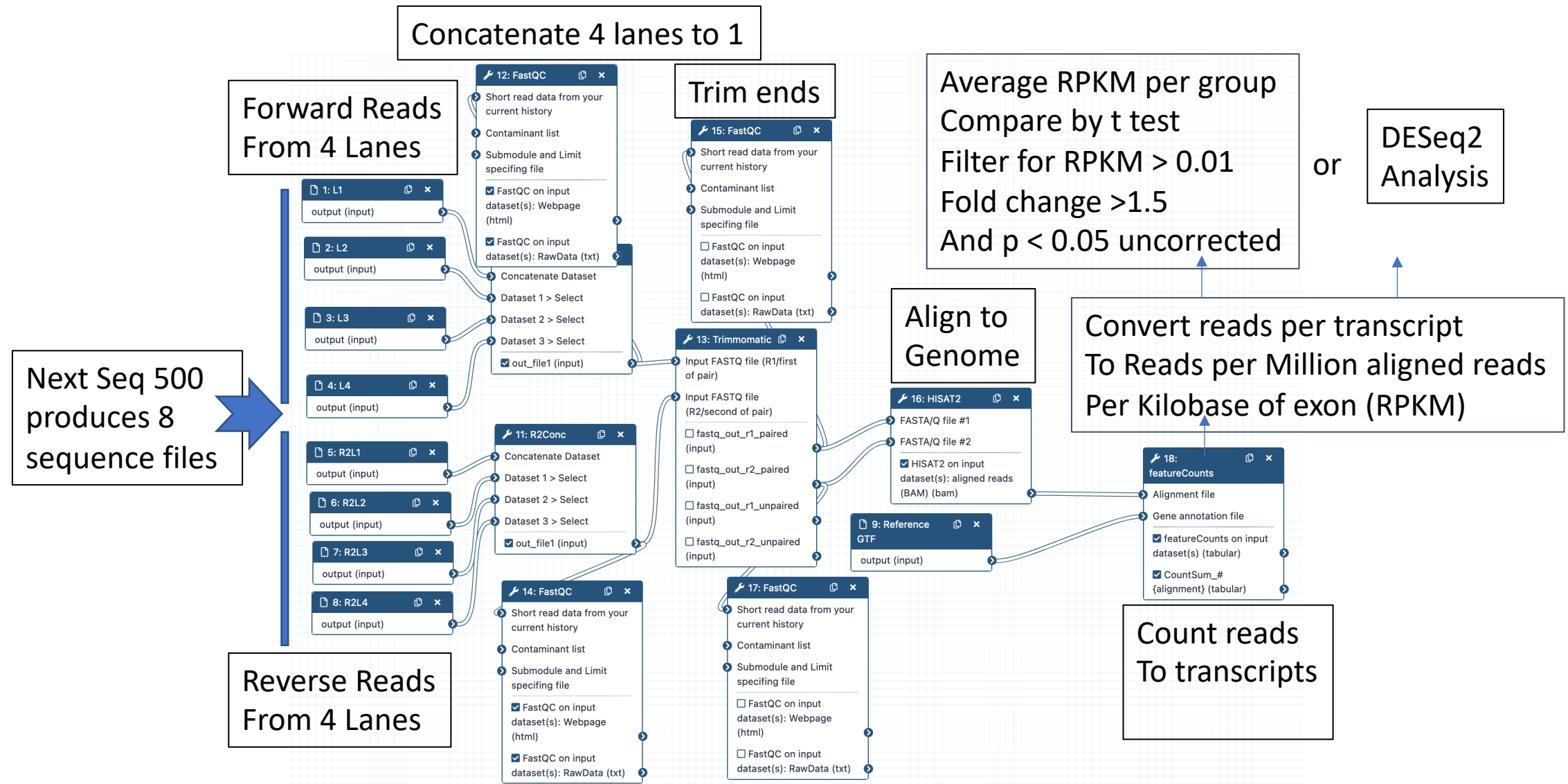

Supplement: Supplementary data 1 — Analytical workflow for RNAseq of human blood RNA [file mmc1.pdf]

SeqLL vs Illumina Base TPM per transcript

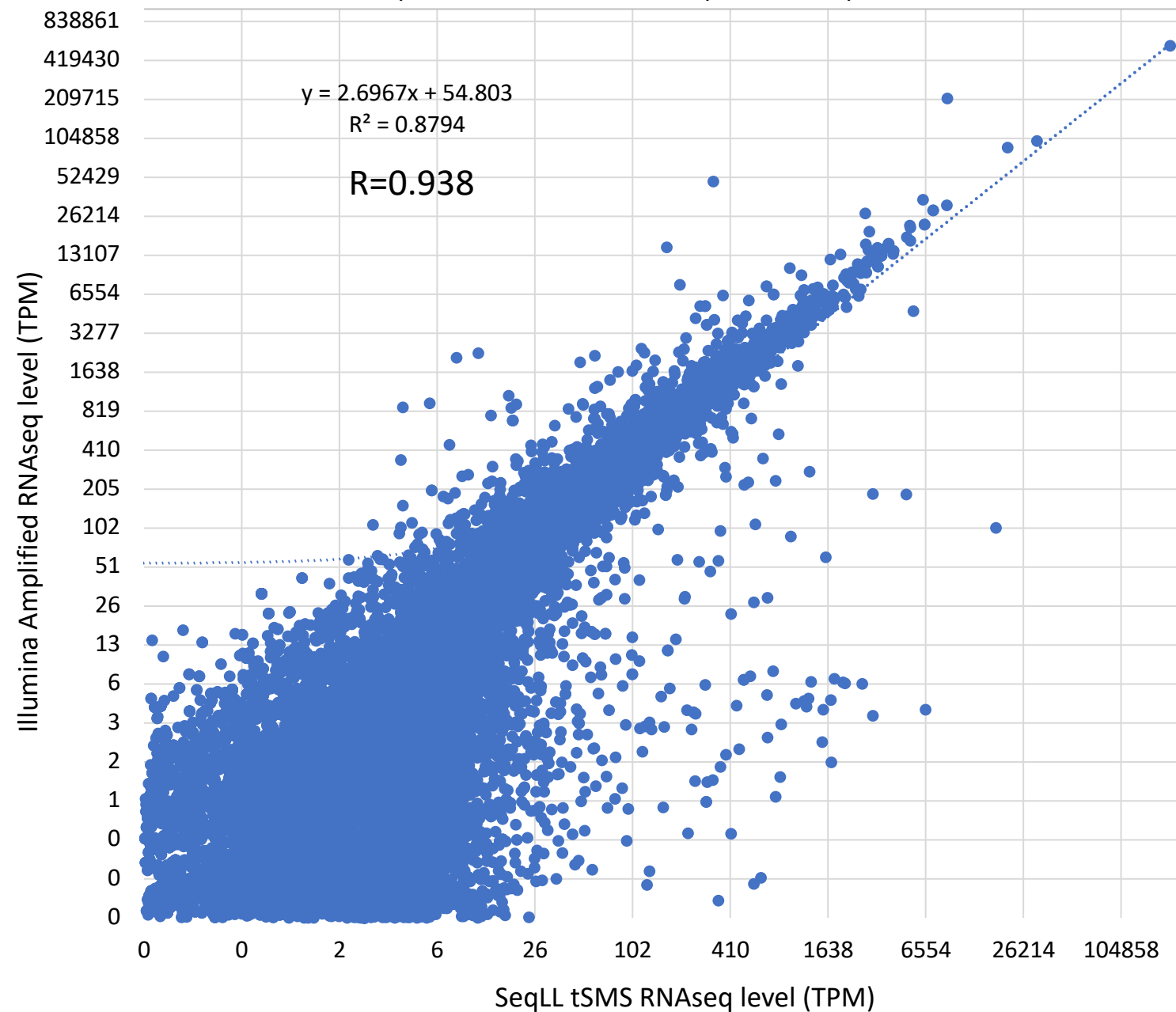

Sup Data 2

Supplement: Supplementary Data 2 — Correlation of SeqLL vs Illumina baseline TPM per transcript. [file mmc2.pdf]

# Supplementary Data 5. Illumina run to run variation by RPKM

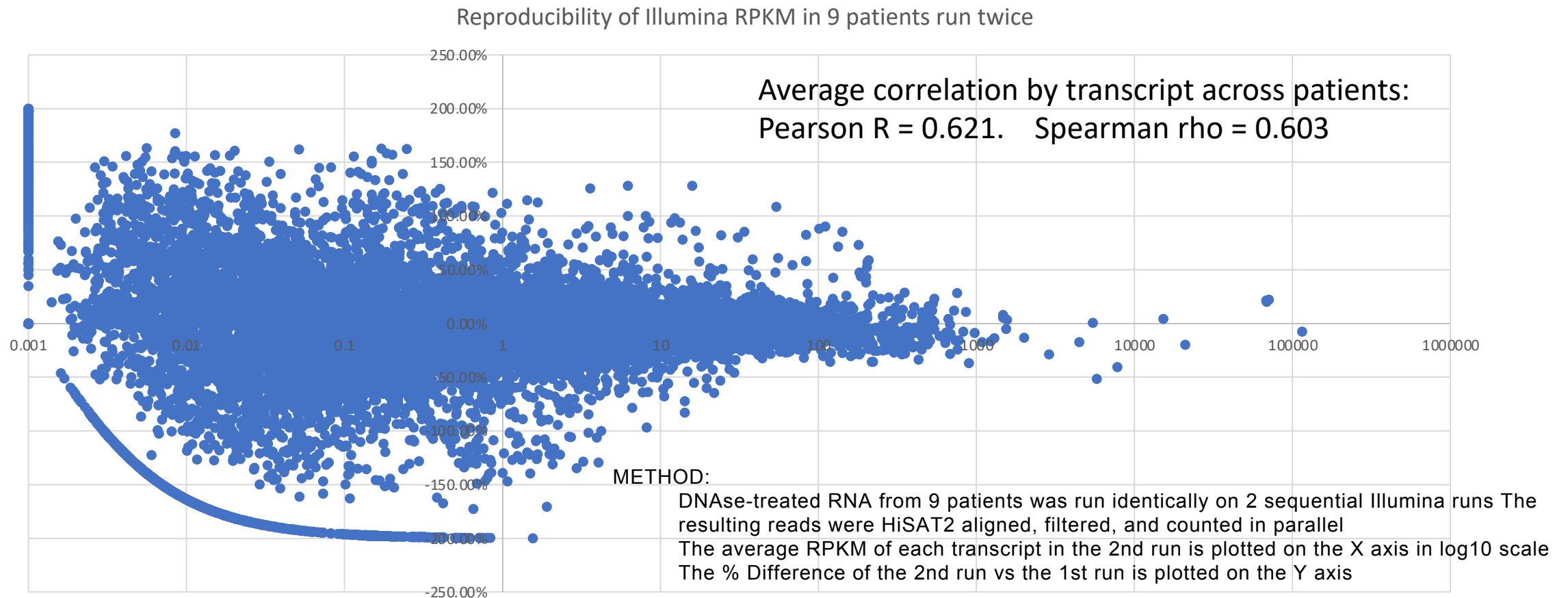

Supplement: Supplementary Data 5 — Illumina run to run variation by RPKM [file mmc5.pdf]
