# Supplementary material for: RNAseq profiling of blood from patients with coronary artery disease: Signature of a T cell imbalance
Source: J Mol Cell Cardiol Plus. 2023 Mar 25;4:100033. doi: 10.1016/j.jmccpl.2023.100033 (PMC10256136; doi:10.1016/j.jmccpl.2023.100033)
Supplement: Supplementary Data 3 — Illumina DEG transcripts annotated (177 patients) [file mmc3.pdf]

| Gene Symbol | Description | Expression baseMean | Expression log2FoldChai | Expression Abs Fold Up | Expression Fold Down | DESeq2 p value | DESeq2 p adjusted | Mann-Whitney P-value | adj. P-value for 102 genes | adj. P-value for 28K genes |
|---|---|---|---|---|---|---|---|---|---|---|
| **UP-REGULATED IN CAD** | | | | | | | | | | |
| FMOD | fibromodulin (from HGNC FMOD) | 0.464 | 1.499 | 2.827 | | 0.042 | 0.99996 | 0.04400 | 0.09169216 | >0.9 |
| BOC | BOC cell adhesion associated, oncogene regulated (from HGNC BOC) | 0.435 | 1.203 | 2.302 | | 0.007 | 0.99996 | 0.04540 | 0.09169216 | >0.9 |
| ADAM29 | ADAM metallopeptidase domain 29 (ADAM29), transcript variant 7, mRNA. ( | 0.579 | 1.167 | 2.245 | | 0.037 | 0.99996 | 0.87500 | 0.88375000 | >0.9 |
| LINC01037 | Long Intergenic Non-Protein Coding RNA 1037 | 0.554 | 1.111 | 2.161 | | 0.002 | 0.99996 | 0.01110 | 0.04484400 | >0.9 |
| MRAP2 | melanocortin 2 receptor accessory protein 2 (MRAP2), transcript variant 1, m | 0.404 | 1.074 | 2.106 | | 0.023 | 0.99996 | 0.18600 | 0.23482500 | >0.9 |
| TEAD1 | TEA domain transcription factor 1 (TEAD1), mRNA. (from RefSeq NM_02196 | 0.758 | 1.063 | 2.089 | | 0.004 | 0.99996 | 0.07640 | 0.12445806 | >0.9 |
| PIGR | polymeric immunoglobulin receptor (from HGNC PIGR)\|\|polymeric immuno | 1.274 | 1.055 | 2.077 | | 0.011 | 0.99996 | 0.20600 | 0.25067470 | >0.9 |
| TMEM98 | Transmembrane Protein 98\| The sequence shown here is derived from an Er | 1.007 | 1.052 | 2.073 | | 0.010 | 0.99996 | 0.14800 | 0.19930667 | >0.9 |
| FAM9C | family with sequence similarity 9 member C (from HGNC FAM9C)\|\|family w | 0.576 | 1.038 | 2.053 | | 0.004 | 0.99996 | 0.05800 | 0.10460714 | >0.9 |
| LOC100996720 | uncharacterized LOC100996720 (LOC100996720), long non-coding RNA. (fror | 0.546 | 1.005 | 2.007 | | 0.015 | 0.99996 | 0.04080 | 0.09169216 | >0.9 |
| LINC02506 | long intergenic non-protein coding RNA 2506 (from HGNC LINC02506)\|\|long | 5.472 | 0.999 | 1.998 | | 0.046 | 0.99996 | 0.82200 | 0.84716327 | >0.9 |
| KCNG2 | potassium voltage-gated channel modifier subfamily G member 2 (from HG | 0.652 | 0.991 | 1.987 | | 0.031 | 0.99996 | 0.98900 | 0.98900000 | >0.9 |
| DCDC2B | doublecortin domain containing 2B (from HGNC DCDC2B)\|\|doublecortin don | 0.470 | 0.990 | 1.986 | | 0.004 | 0.99996 | 0.00618 | 0.04390133 | >0.9 |
| GLDN | gliomedin (GLDN), transcript variant 2, mRNA. (from RefSeq NM_00133029 | 0.718 | 0.984 | 1.978 | | 0.020 | 0.99996 | 0.46400 | 0.50939130 | >0.9 |
| MIR548A1HG | MIR548A1 host gene (from HGNC MIR548A1HG)\|\|MIR548A1 host gene (MI | 0.423 | 0.961 | 1.946 | | 0.011 | 0.99996 | 0.03510 | 0.08843659 | >0.9 |
| DUXB | double homeobox B (DUXB), transcript variant 3, mRNA. (from RefSeq NM_( | 1.678 | 0.941 | 1.919 | | 0.001 | 0.99996 | 0.04630 | 0.09169216 | >0.9 |
| MSLN | mesothelin (MSLN), transcript variant 2, mRNA. (from RefSeq NM_013404) | 0.914 | 0.933 | 1.910 | | 0.048 | 0.99996 | 0.10000 | 0.15074627 | >0.9 |
| FLOT1 | flotillin 1 (from HGNC FLOT1) | 5.976 | 0.928 | 1.903 | | 0.007 | 0.99996 | 0.01250 | 0.04855769 | >0.9 |
| MIR1277 | microRNA 1277 (MIR1277), microRNA. (from RefSeq NR_031685)\|\|microRI | 0.572 | 0.908 | 1.876 | | 0.004 | 0.99996 | 0.00528 | 0.04390133 | >0.9 |
| CT75 | CT75 (from geneSymbol)\|\|cancer/testis associated transcript 75 (CT75), tra | 0.484 | 0.880 | 1.840 | | 0.015 | 0.99996 | 0.01810 | 0.06093667 | >0.9 |
| OVCH1-AS1 | OVCH1 antisense RNA 1 (from HGNC OVCH1-AS1)\|\|OVCH1 antisense RNA 1 | 11.272 | 0.842 | 1.793 | | 0.009 | 0.99996 | 0.24100 | 0.27978161 | >0.9 |
| HBBP1 | hemoglobin subunit beta pseudogene 1 (from HGNC HBBP1)\|\|hemoglobin s | 0.821 | 0.832 | 1.780 | | 0.046 | 0.99996 | 0.30700 | 0.34839326 | >0.9 |
| RAP1GAP | RAP1 GTPase activating protein (from HGNC RAP1GAP) | 99.459 | 0.827 | 1.774 | | 0.013 | 0.99996 | 0.54000 | 0.57128125 | >0.9 |
| DUOX1 | dual oxidase 1 (from HGNC DUOX1)\|\|dual oxidase 1 (DUOX1), transcript var | 0.719 | 0.820 | 1.765 | | 0.008 | 0.99996 | 0.01050 | 0.04484400 | >0.9 |
| LYPD1 | LY6/PLAUR Domain Containing 1 | 0.468 | 0.818 | 1.763 | | 0.021 | 0.99996 | 0.01010 | 0.04484400 | >0.9 |
| LINC01251 | long intergenic non-protein coding RNA 1251 (LINC01251), long non-coding F | 0.375 | 0.798 | 1.739 | | 0.029 | 0.99996 | 0.13600 | 0.18816438 | >0.9 |
| FN1 | fibronectin 1 (from HGNC FN1)\|\|fibronectin 1 (FN1), transcript variant 4, m | 10.588 | 0.793 | 1.733 | | 0.002 | 0.99996 | 0.41900 | 0.46504396 | >0.9 |
| MTRNR2L1 | MT-RNR2 like 1 (MTRNR2L1), mRNA. (from RefSeq NM_001190452)\|\|MT-F | 57.174 | 0.779 | 1.716 | | 0.039 | 0.99996 | 0.17700 | 0.22629114 | >0.9 |
| FAP | fibroblast activation protein alpha (from HGNC FAP)\|\|fibroblast activation p | 1.005 | 0.773 | 1.709 | | 0.013 | 0.99996 | 0.23500 | 0.27598837 | >0.9 |
| BTN1A1 | butyrophilin subfamily 1 member A1 (BTN1A1), mRNA. (from RefSeq NM_0 | 0.700 | 0.773 | 1.708 | | 0.004 | 0.99996 | 0.0157 | 0.05522069 | >0.9 |
| KCNJ11 | potassium voltage-gated channel subfamily J member 11 (from HGNC KCNJ1 | 0.682 | 0.768 | 1.703 | | 0.034 | 0.99996 | 0.08390 | 0.13240469 | >0.9 |
| LOC100507144 | uncharacterized LOC100507144 (LOC100507144), long non-coding RNA. (fror | 0.513 | 0.767 | 1.702 | | 0.017 | 0.99996 | 0.04260 | 0.09169216 | >0.9 |
| MIR8075 | microRNA 8075 (MIR8075), microRNA. (from RefSeq NR_107042)\|\|microRI | 0.543 | 0.761 | 1.694 | | 0.031 | 0.99996 | 0.05620 | 0.10320364 | >0.9 |
| MIR4440 | microRNA 4440 (MIR4440), microRNA. (from RefSeq NR_039642)\|\|microRI | 0.789 | 0.750 | 1.682 | | 0.010 | 0.99996 | 0.09820 | 0.15027576 | >0.9 |
| TUBB | tubulin beta class I (from HGNC TUBB) | 11.711 | 0.738 | 1.668 | | 0.037 | 0.99996 | 0.03010 | 0.08216486 | >0.9 |
| SLC7A10 | solute carrier family 7 member 10 (from HGNC SLC7A10)\|\|solute carrier far | 0.517 | 0.734 | 1.664 | | 0.041 | 0.99996 | 0.22000 | 0.26452381 | >0.9 |
| SLCO2A1 | solute carrier organic anion transporter family member 2A1 (SLCO2A1), mRI | 0.817 | 0.728 | 1.656 | | 0.018 | 0.99996 | 0.01770 | 0.06093667 | >0.9 |
| FAM182B | family with sequence similarity 182 member B (from HGNC FAM182B) | 0.638 | 0.726 | 1.654 | | 0.032 | 0.99996 | 0.36600 | 0.41073333 | >0.9 |
| MUC20 | Mucin 20, Cell Surface Associated | 0.940 | 0.723 | 1.650 | | 0.040 | 0.99996 | 0.20600 | 0.25067470 | >0.9 |
| OMP | olfactory marker protein (OMP), mRNA. (from RefSeq NM_006189)\|\|olfacto | 0.427 | 0.716 | 1.642 | | 0.038 | 0.99996 | 0.02530 | 0.07238333 | >0.9 |
| LINC00535 | long intergenic non-protein coding RNA 535 (LINC00535), long non-coding RI | 0.483 | 0.707 | 1.632 | | 0.031 | 0.99996 | 0.02280 | 0.07196250 | >0.9 |
| GIMAP5 | Required for mitochondrial integrity and T-cell survival. May contribute to T- | 0.658 | 0.705 | 1.630 | | 0.020 | 0.99996 | 0.12600 | 0.18180000 | >0.9 |
| LOC101927018 | uncharacterized LOC101927018 (LOC101927018), long non-coding RNA. (fror | 1.134 | 0.693 | 1.617 | | 0.012 | 0.99996 | 0.04310 | 0.09169216 | >0.9 |
| FIGN | fidgetin, microtubule severing factor (from HGNC FIGN)\|\|fidgetin, microtub | 1.739 | 0.680 | 1.603 | | 0.035 | 0.99996 | 0.01030 | 0.04484400 | >0.9 |
| ETV7 | ETS variant transcription factor 7 (ETV7), transcript variant 6, mRNA. (from I | 32.038 | 0.677 | 1.599 | | 0.001 | 0.99996 | 0.26300 | 0.30185227 | >0.9 |
| ADCY1 | adenylate cyclase 1 (from HGNC ADCY1)\|\|adenylate cyclase 1 (ADCY1), tran | 0.459 | 0.672 | 1.593 | | 0.048 | 0.99996 | 0.16300 | 0.21380519 | >0.9 |
| PCOLCE2 | procollagen C-endopeptidase enhancer 2 (from HGNC PCOLCE2)\|\|procollage | 1.284 | 0.667 | 1.588 | | 0.029 | 0.99996 | 0.12800 | 0.18208451 | >0.9 |
| LINC02073 | long intergenic non-protein coding RNA 2073 (from HGNC LINC02073)\|\|long | 3.884 | 0.659 | 1.579 | | 0.017 | 0.99996 | 0.54300 | 0.57128125 | >0.9 |
| C5orf17 | chromosome 5 putative open reading frame 17 (C5orf17), long non-coding R | 2.363 | 0.659 | 1.579 | | 0.036 | 0.99996 | 0.72400 | 0.75385567 | >0.9 |
| MIR12130 | MicroRNA 12130 | 0.600 | 0.656 | 1.575 | | 0.047 | 0.99996 | 0.02120 | 0.06907097 | >0.9 |
| MIR7853 | microRNA 7853 (MIR7853), microRNA. (from RefSeq NR_107007)\|\|microRI | 0.456 | 0.644 | 1.563 | | 0.047 | 0.99996 | 0.05000 | 0.09528302 | >0.9 |
| HTR1B | 5-hydroxytryptamine receptor 1B (HTR1B), mRNA. (from RefSeq NM_00086 | 0.706 | 0.631 | 1.549 | | 0.028 | 0.99996 | 0.07510 | 0.12434590 | >0.9 |
| SPATA45 | spermatogenesis associated 45 (SPATA45), mRNA. (from RefSeq NM_00102 | 0.939 | 0.626 | 1.544 | | 0.012 | 0.99996 | 0.04370 | 0.09169216 | >0.9 |
| LINC01529 | long intergenic non-protein coding RNA 1529 (from HGNC LINC01529)\|\|long | 1.032 | 0.626 | 1.543 | | 0.034 | 0.99996 | 0.49900 | 0.53938298 | >0.9 |
| WFIKKN1 | WAP, follistatin/kazal, immunoglobulin, kunitz and netrin domain containing | 1.164 | 0.625 | 1.542 | | 0.019 | 0.99996 | 0.14500 | 0.19790541 | >0.9 |
| TLL2 | tolloid like 2 (from HGNC TLL2)\|\|tolloid like 2 (TLL2), mRNA. (from RefSeq N | 0.652 | 0.622 | 1.539 | | 0.036 | 0.99996 | 0.16200 | 0.21380519 | >0.9 |
| BMPR1B | bone morphogenetic protein receptor type 1B (BMPR1B), transcript variant 1 | 0.766 | 0.607 | 1.523 | | 0.038 | 0.99996 | 0.17400 | 0.22530769 | >0.9 |
| NME9 | NME/NM23 family member 9 (NME9), transcript variant 2, mRNA. (from Re | 0.930 | 0.600 | 1.516 | | 0.016 | 0.99996 | 0.07500 | 0.12434590 | >0.9 |
| ERFE | erythroferrone (ERFE), mRNA. (from RefSeq NM_001291832)\|\|erythroferro | 2.674 | 0.598 | 1.514 | | 0.021 | 0.99996 | 0.19200 | 0.23940741 | >0.9 |
| SNORA117 | Small Nucleolar RNA, H/ACA Box 117 | 1.037 | 0.597 | 1.512 | | 0.012 | 0.99996 | 0.04620 | 0.09169216 | >0.9 |
| LRRC32 | leucine rich repeat containing 32 (from HGNC LRRC32)\|\|leucine rich repeat | 2.057 | 0.587 | 1.502 | | 0.004 | 0.99996 | 0.08010 | 0.12841429 | >0.9 |
| **DOWN-REGULATED IN CAD** | | | | | | | | | | |
| ECHDC3 | enoyl-CoA hydratase domain containing 3 (from HGNC ECHDC3)\|\|enoyl-CoA | 30.008 | -0.600 | 0.660 | 1.516 | 0.003 | 0.9999641 | 0.22800 | 0.27091765 | >0.9 |
| LINC02575 | long intergenic non-protein coding RNA 2575 (LINC02575), long non-coding F | 0.707 | -0.602 | 0.659 | 1.518 | 0.041 | 0.9999641 | 0.04800 | 0.09323077 | >0.9 |
| MIR4681 | microRNA 4681 (MIR4681), microRNA. (from RefSeq NR_039829)\|\|microRI | 0.642 | -0.611 | 0.655 | 1.527 | 0.025 | 0.9999641 | 0.00500 | 0.04390133 | >0.9 |
| SMKR1 | small lysine rich protein 1 (from HGNC SMKR1)\|\|small lysine rich protein 1 ( | 1.886 | -0.612 | 0.654 | 1.528 | 0.001 | 0.9999641 | 0.00456 | 0.04390133 | >0.9 |
| STRC | stereocilin (STRC), mRNA. (from RefSeq NM_153700) | 0.713 | -0.619 | 0.651 | 1.536 | 0.025 | 0.9999641 | 0.00913 | 0.04484400 | >0.9 |
| LINC02139 | long intergenic non-protein coding RNA 2139 (LINC02139), long non-coding F | 0.762 | -0.624 | 0.649 | 1.541 | 0.020 | 0.9999641 | 0.00946 | 0.04484400 | >0.9 |
| LRRC7 | leucine rich repeat containing 7 (LRRC7), transcript variant 11, mRNA. (from | 9.036 | -0.631 | 0.646 | 1.548 | 0.010 | 0.9999641 | 0.13300 | 0.18656944 | >0.9 |
| CLIC6 | chloride intracellular channel 6 (CLIC6), transcript variant 1, mRNA. (from Re | 0.712 | -0.634 | 0.644 | 1.552 | 0.033 | 0.9999641 | 0.03230 | 0.08585000 | >0.9 |
| NRCAM | neuronal cell adhesion molecule (from HGNC NRCAM) | 7.903 | -0.636 | 0.643 | 1.554 | 0.004 | 0.9999641 | 0.02580 | 0.07238333 | >0.9 |
| LINC01393 | long intergenic non-protein coding RNA 1393 (from HGNC LINC01393)\|\|long | 0.575 | -0.647 | 0.639 | 1.565 | 0.034 | 0.9999641 | 0.01470 | 0.05498889 | >0.9 |
| LOC101928438 | uncharacterized LOC101928438 (LOC101928438), long non-coding RNA. (fror | 0.673 | -0.648 | 0.638 | 1.567 | 0.028 | 0.9999641 | 0.00485 | 0.04390133 | >0.9 |
| EFCC1 | EF-hand and coiled-coil domain containing 1 (EFCC1), transcript variant 1, m | 0.557 | -0.651 | 0.637 | 1.570 | 0.030 | 0.9999641 | 0.03460 | 0.08843659 | >0.9 |
| LOC100996671 | uncharacterized LOC100996671 (LOC100996671), transcript variant 3, long n | 1.010 | -0.654 | 0.636 | 1.573 | 0.023 | 0.9999641 | 0.01110 | 0.04484400 | >0.9 |
| LENG8 | leukocyte receptor cluster member 8 (LENG8), transcript variant 3, mRNA. (f | 1.072 | -0.663 | 0.631 | 1.584 | 0.009 | 0.9999641 | 0.00947 | 0.04484400 | >0.9 |
| MYCBP2-AS1 | MYCBP2 antisense RNA 1 (from HGNC MYCBP2-AS1)\|\|MYCBP2 antisense RI | 0.968 | -0.683 | 0.623 | 1.606 | 0.021 | 0.9999641 | 0.05540 | 0.10320364 | >0.9 |
| RN7SL3 | RNA component of signal recognition particle 7SL3 (RN7SL3), small cytoplas | 13.253 | -0.686 | 0.622 | 1.609 | 0.000 | 0.9999641 | 0.50200 | 0.53938298 | >0.9 |
| GPRC5D | G protein-coupled receptor class C group 5 member D (GPRC5D), mRNA. (frc | 0.966 | -0.718 | 0.608 | 1.645 | 0.029 | 0.9999641 | 0.00519 | 0.04390133 | >0.9 |
| RNVU1-1 | RNA, variant U1 small nuclear 1 (RNVU1-1), small nuclear RNA. (from RefSe | 0.622 | -0.731 | 0.602 | 1.660 | 0.038 | 0.9999641 | 0.10500 | 0.15595588 | >0.9 |
| RNY3 | RNA, Ro60-associated Y3 (RNY3), Y RNA. (from RefSeq NR_004392)\|\|RNA, | 0.579 | -0.736 | 0.600 | 1.666 | 0.036 | 0.9999641 | 0.06610 | 0.11510517 | >0.9 |
| NUTM2B | NUT family member 2B (NUTM2B), mRNA. (from RefSeq NM_001278495)\| | 1.041 | -0.737 | 0.600 | 1.667 | 0.003 | 0.9999641 | 0.00944 | 0.04484400 | >0.9 |
| EIF2S3B | eukaryotic translation initiation factor 2 subunit gamma B (EIF2S3B), transcr | 8.680 | -0.738 | 0.600 | 1.667 | 0.020 | 0.9999641 | 0.03590 | 0.08843659 | >0.9 |
| SIAH3 | siah E3 ubiquitin protein ligase family member 3 (SIAH3), mRNA. (from RefS | 0.847 | -0.748 | 0.596 | 1.679 | 0.004 | 0.9999641 | 0.00325 | 0.04390133 | >0.9 |
| BHLHA15 | basic helix-loop-helix family member a15 (BHLHA15), mRNA. (from RefSeq I | 0.950 | -0.753 | 0.593 | 1.685 | 0.033 | 0.9999641 | 0.12500 | 0.18180000 | >0.9 |
| LOC105379143 | uncharacterized LOC105379143 (LOC105379143), transcript variant 2, long n | 0.874 | -0.754 | 0.593 | 1.686 | 0.006 | 0.9999641 | 0.04550 | 0.09169216 | >0.9 |
| MARCHF10 | membrane associated ring-CH-type finger 10 (MARCHF10), transcript variant | 0.561 | -0.755 | 0.593 | 1.687 | 0.011 | 0.9999641 | 0.00493 | 0.04390133 | >0.9 |
| TAS2R46 | taste 2 receptor member 46 (TAS2R46), mRNA. (from RefSeq NM_176887) | 1.245 | -0.764 | 0.589 | 1.698 | 0.004 | 0.9999641 | 0.00780 | 0.04484400 | >0.9 |
| SFRP5 | secreted frizzled related protein 5 (SFRP5), mRNA. (from RefSeq NM_00301 | 0.751 | -0.772 | 0.586 | 1.707 | 0.038 | 0.9999641 | 0.00652 | 0.04390133 | >0.9 |
| KCNA5 | potassium voltage-gated channel subfamily A member 5 (KCNA5), mRNA. (f | 0.796 | -0.775 | 0.584 | 1.712 | 0.003 | 0.9999641 | 0.00105 | 0.04390133 | >0.9 |
| LOC101928932 | uncharacterized LOC101928932 (LOC101928932), long non-coding RNA. (fror | 0.663 | -0.783 | 0.581 | 1.721 | 0.024 | 0.9999641 | 0.06930 | 0.11863220 | >0.9 |
| SCGB3A1 | secretoglobin family 3A member 1 (from HGNC SCGB3A1)\|\|secretoglobin fa | 1.185 | -0.792 | 0.578 | 1.731 | 0.028 | 0.9999641 | 0.01570 | 0.05663214 | >0.9 |
| TXNDC5 | thioredoxin domain containing 5 (from HGNC TXNDC5)\|\|thioredoxin domain | 1.243 | -0.798 | 0.575 | 1.739 | 0.002 | 0.9999641 | 0.02520 | 0.07238333 | >0.9 |
| LOC100507002 | uncharacterized LOC100507002 (LOC100507002), long non-coding RNA. (fror | 0.487 | -0.844 | 0.557 | 1.795 | 0.013 | 0.9999641 | 0.02380 | 0.07238333 | >0.9 |
| CNTNAP3P2 | CNTNAP3 pseudogene 2 (CNTNAP3P2), non-coding RNA. (from RefSeq NR_1 | 2.467 | -0.845 | 0.557 | 1.797 | 0.020 | 0.9999641 | 0.86200 | 0.87941414 | >0.9 |
| APBA2 | amyloid beta precursor protein binding family A member 2 (APBA2), transcri | 1.407 | -0.868 | 0.548 | 1.825 | 0.009 | 0.9999641 | 0.00446 | 0.04390133 | >0.9 |
| GUCY1B2 | guanylate cyclase 1 soluble subunit beta 2 (pseudogene) (from HGNC GUCY1 | 1.005 | -0.931 | 0.524 | 1.907 | 0.049 | 0.9999641 | 0.06550 | 0.11510517 | >0.9 |
| SHISA2 | shisa family member 2 (SHISA2), mRNA. (from RefSeq NM_001007538)\|\|sh | 1.027 | -0.965 | 0.512 | 1.952 | 0.001 | 0.9999641 | 0.00153 | 0.04390133 | >0.9 |
| STK19 | serine/threonine kinase 19 (STK19), transcript variant 1, mRNA. (from RefSe | 0.437 | -0.980 | 0.507 | 1.972 | 0.011 | 0.9999641 | 0.00362 | 0.04390133 | >0.9 |
| ZNRD1 | RNA Polymerase I Subunit H , Zinc Ribbon Domain Containing 1 | 3.309 | -1.022 | 0.493 | 2.030 | 0.030 | 0.9999641 | 0.04210 | 0.09169216 | >0.9 |
| REEP1 | receptor accessory protein 1 (from HGNC REEP1)\|\|receptor accessory protei | 2.927 | -1.023 | 0.492 | 2.032 | 0.000 | 0.9999641 | 0.00180 | 0.04390133 | >0.9 |
| PNMA8A | PNMA family member 8A (from HGNC PNMA8A)\|Paraneoplastic Ma Antigei | 0.413 | -1.168 | 0.445 | 2.248 | 0.029 | 0.9999641 | 0.00577 | 0.04390133 | >0.9 |
| NEBL | nebulette (from HGNC NEBL)\|\|nebulette (NEBL), transcript variant 2, mRNA | 41.831 | -1.249 | 0.421 | 2.376 | 0.000 | 0.9999641 | 0.09690 | 0.15027576 | >0.9 |

| Gene Symbol | AKA | Pathway | Interaction | Cell type | Function/Disease |
|---|---|---|---|---|---|
| **UP-REGULATED IN CAD** | | | | | |
| FMOD | | TGF | | T cells | Athero Ferroptosis |
| BOC | Brother of CDO | hedgehog | CDO Hedgehog | | |
| ADAM29 | | | | human testis (exclus) | signal transduction during |
| LINC01037 | - | | | - | - |
| MRAP2 | | ACTH | Melanocortin 4 Rece | adrenal glucocorticoid | obesity |
| TEAD1 | YAP1 | Hippo | | memory CD4+ Tcell | proatherogenic phenotype |
| PIGR | | liver fibrosis | | epithelial | mucosal immunity |
| TMEM98 | | atherosclerosis | Wnt/catenin | Th1 differentiation | Athero |
| FAM9C | | PI3/Akt pathway | | testis (exclus) | overexpression increased |
| LOC100996720 | - | - | | - | - |
| LINC02506 | - | cardiac hypertrophy | | - | regulators of genetic |
| KCNG2 | - | BMP-2 (which down | | - | affects the function of Kv |
| DCDC2B | - | | TUBB (2B) | hair cells kinocilia | primary cilia length |
| GLDN | - | Nodal | NRCAM | olfactomedin-like and co | promotes nodes of Ranvier |
| MIR548A1HG | | | TLR | Immune | Athero |
| DUXB | | | | | |
| MSLN | megakaryocyte | potentiating factor (M | steriocilin | megakaryocte, Treg | major neoantigen in cancer |
| FLOT1 | | | | CD8+ T cell differentiation | |
| MIR1277 | - | - | | - | - |
| CT75 | - | - | | testis | RNA Gene, and is affiliated |
| OVCH1-AS1 | ncRNA | - | - | liver cells | Hepatocellular Carcinoma |
| HBBP1 | LINC00304 | | | | γ-globin production |
| RAP1GAP | | | | | Athero |
| DUOX1 | - | NADPH oxidases | | respiratory epithelial cell | knockdown of DUOX1 |
| LYPD1 | - | ASD, Breast/ovarian | | heart fibroblasts, synapti | acetylcholine receptor |
| LINC01251 | | | | | |
| FN1 | | Aortic Valve | | T cells affected | couples cardiac myocytes |
| MTRNR2L1 | HN1, humanin-l | liver parenchyma, | | | correlation with insulin |
| FAP | | | | | cardiac wound healing |
| BTN1A1 | | BTN1A1, bound to | | T cells affected | Inhibits the proliferation of |
| KCNJ11 | - | | | | type 2 diabetes weak |
| LOC100507144 | lncRNA | | | | |
| MIR8075 | miR-8075 | regulatory function | | regulatory mechanisms ( | post-transcriptional |
| MIR4440 | | | | | |
| TUBB | - | CAD, brain | | B-cells | Athero GWAS |
| SLC7A10 | ASC1 | | | synaptic | |
| SLCO2A1 | prostaglandin tı | prostaglandin | | the uptake and clearance | of prostaglandins in numero |
| FAM182B | lncRNA | | | | |
| MUC20 | - | endometrium, | | middle ear epithelium, c | sperm maturation in |
| OMP | - | ATP showed the bell- | | | modulator within the |
| LINC00535 | | | | | |
| GIMAP5 | IAN5 | | | CD4+ T cell | |
| LOC101927018 | lncRNA | PI3K-Akt | | | - |
| FIGN | | | | | microtubule, synaptic |
| ETV7 | Ets variant 7 | cell apoptosis and | | CD8+ T cell, Infiltration o | promoted infiltration of |
| ADCY1 | - | Asiaticoside/ | | Glomerular podocytes, n | autosomal recessive |
| PCOLCE2 | - | gastic, thyroid | | | |
| LINC02073 | lncRNA | | | | |
| C5orf17 | LINC02899 | - | - | lungs squamous cell carc | typical lncRNA |
| MIR12130 | | | | | |
| MIR7853 | microRNA | - | - | - | - |
| HTR1B | | | | | release of serotonin, |
| SPATA45 | C1orf227 | | | | |
| LINC01529 | | | | | |
| WFIKKN1 | | GDF | | GDF path | |
| TLL2 | | GDF, BMP | | GDF BMP | |
| BMPR1B | - | BMP TGF | BMP ligands | reproductive system | |
| NME9 | Contains 1 thioredoxin domain | | | | |
| ERFE | FAM132B | (-) gluconeogenesis | | | enable hormone activity |
| SNORA117 | - | - | - | - | snoRNAs |
| LRRC32 | GARP, Transforming Growth Factor Beta Activator | | | Treg | Athero |
| **DOWN-REGULATED IN CAD** | | | | | |
| ECHDC3 | - | Fatty acid | enable enoyl-CoA hy | platelet | insulin-stimulated glucose |
| LINC02575 | lncRNA | - | - | LSCC (laryngeal squamou | proliferation, migration, |
| MIR4681 | - | - | - | - | - |
| SMKR1 | - | epigenetics/methyla | - | testis (exclus), brain | - |
| STRC | DFNB16 | hearing loss | connects to mesothe | stereocilia of the sensory | - |
| LINC02139 | - | - | - | | - |
| LRRC7 | densin-180 | apical/basal polarity | glutamatergic posts | synaptic, epithelial cell | critical regulator of |
| CLIC6 | - | (3!) Neuropathic | - | stomach, retinal pigment | cell |
| NRCAM | - | - | gliomedin, | CD4+ T cell | adhesion molecules |
| LINC01393 | lncRNA | - | - | - | - |
| LOC101928438 | lncRNA | - | - | - | - |
| EFCC1 | - | - | - | - | enable calcium ion binding |
| LOC100996671 | - | - | - | - | - |
| LENG8 | pp13842 | Pemphigus foliaceus | - | leukocyte, autoimmune ( | - |
| MYCBP2-AS1 | - | - | - | - | - |
| RN7SL3 | 7L30.2; RNSRP3 | - | - | - | mediates cotranslational |
| GPRC5D | - | DuoBody antibody | DuoBody antibody | plasma cells, B-cell matu | ligand and signaling |
| RNVU1-1 | snRNA | - | - | - | - |
| RNY3 | YRNA, Y3, HY3 | downregulated in | | platelet? | manages proportion of S |
| NUTM2B | FAM22B, bA119 | - | - | - | - |
| EIF2S3B | EIF2, EIF2G, ME | - | - | testis | largest subunit of a |
| SIAH3 | - | pathogenesis of | - | - | increased rate of |
| BHLHA15 | MIST1, BHLHB8 | antibody secretory | co-operate with XBP | plasma cell, secretory ep | induce and maintain |
| LOC105379143 | ncRNA | - | - | - | - |
| MARCHF10 | - | Ciliary Dyskinesia | - | - | - |
| TAS2R46 | - | Olfactory Signaling | - | - | expressed on the surface of |
| SFRP5 | SARP3 | Wnt, obesity | - | white adipose tissue | relationship between Sfrp5 |
| KCNA5 | - | Antiarrhythmic | - | Cardiac Potassium Chann | atrial electrical activity, |
| LOC101928932 | lncRNA | - | - | - | - |
| SCGB3A1 | HIN1 | (+) reg of myoblast | IL-1β-TNFα | myoblast, lung epithelia | Alz. D risk genes, tumor |
| TXNDC5 | HCC2 | angiogenesis | - | - | catalyze protein folding |
| LOC100507002 | lncRNA | - | - | colon | |
| CNTNAP3P2 | pseudo | - | - | esophagus | |
| APBA2 | MINT2, X11L | - | amyloid precursor | synaptic adaptor protein | stabilizes APP and inhibits |
| GUCY1B2 | - | Endothelin pathways, | - | - | - |
| SHISA2 | - | Wnt signaling | N1ICD inhibited Shi | - | - |
| STK19 | - | Melanoma | | | DNA repair |
| ZNRD1 | POLR1H | RNA Polymerase I | - | - | gene expression |
| REEP1 | - | Olfactory Signaling | - | - | - |
| PNMA8A | - | Oligodendroglioma | - | - | - |
| NEBL | Lasp-2 | dilated | binds actin and inter | cardiac muscle | it stretches along the thin |

**NOTES:**
No transcripts were normally distributed according to the Kolmogorov-Smirnov test or the Shaparo-Wilk Test.
Mann-Whitney p value adjusted for multiple testing by FDR
DESEQ2 uses a Benjamini-Hochberg correction for multiple testing
