# Supplementary material for: RNAseq profiling of blood from patients with coronary artery disease: Signature of a T cell imbalance
Source: J Mol Cell Cardiol Plus. 2023 Mar 25;4:100033. doi: 10.1016/j.jmccpl.2023.100033 (PMC10256136; doi:10.1016/j.jmccpl.2023.100033)
Supplement: Supplementary file 3 [file mmc4.docx]

T cells						n=38		n=19		n=25		Low vs
						LOW	LOW	MID		CAD		CAD
						Mean	s.e.m.	Mean	s.e.m.	Mean	s.e.m.	T test
Gene	Description	Cell lineage	nTPM	Specificity score								
FANK1	fibronectin type III and ankyrin repeat domains 1	Detected in	61.2	612		1.060	0.097	0.719	0.147	0.928	0.105	0.371
CTLA4	cytotoxic T-lymphocyte associated protein 4	Detected in	44.5	445		4.684	0.361	4.242	0.449	4.344	0.405	0.541
FOXP3	forkhead box P3	Detected in	53.3	394		2.489	0.240	2.378	0.342	2.621	0.361	0.752
ELOVL4	ELOVL fatty acid elongase 4	Detected in	23.7	238		1.198	0.119	0.771	0.157	0.919	0.139	0.136
PRSS35	serine protease 35	Detected in	19.5	195		0.461	0.097	0.209	0.055	0.259	0.076	0.136
CD248	CD248 molecule	Detected in	21.8	144		0.766	0.115	0.739	0.172	0.466	0.079	0.060
NEFL	neurofilament light	Detected in	11.2	113		2.733	0.348	1.742	0.263	2.156	0.319	0.254
CPA5	carboxypeptidase A5	Detected in	56.4	102		0.463	0.114	0.176	0.072	0.301	0.071	0.294
REG4	regenerating family member 4	Detected in	24.6	101		0.109	0.018	0.142	0.059	0.086	0.024	0.430
TMEM171	transmembrane protein 171	Detected in	24.1	89		0.519	0.83	0.454	0.142	0.355	0.81	0.183
THEMIS	thymocyte selection associated	Detected in	58.3	88		0.002	0.002	0	0	0	0	0.422
PMCH	pro-melanin concentrating hormone	Detected in	9.5	85		0.051	0.025	0.031	0.031	0	0	0.104
					Average	1.211		0.967		1.036		
					T test			0.0139657		0.0075854		

T regulatory cells						n=38		n=19		n=25		Low vs
						LOW	LOW	MID		CAD		CAD
						Mean	s.e.m.	Mean	s.e.m.	Mean	s.e.m.	T test
Gene	Description	Cell type distribution	nTPM	Specificity score								
TRIM16	tripartite motif containing 16	Detected in many	45.1	9		2.592	0.191	2.458	0.280	2.513	0.235	0.796
SEMA3G	semaphorin 3G	Detected in single	1.4	15		0.398	0.034	0.289	0.046	0.350	0.031	0.333
RTKN2	rhotekin 2	Detected in some	16.7	8		3.944	0.362	3.183	0.447	3.268	0.449	0.245
PMCH	pro-melanin concentrating hormone	Detected in some	9.5	10		0.051	0.025	0.031	0.031	0.000	0.000	0.104
OGN	osteoglycin	Detected in single	1.7	9		0.117	0.020	0.105	0.028	0.134	0.035	0.653
FOXP3	forkhead box P3	Detected in some	53.3	11		2.489	0.240	2.378	0.342	2.621	0.361	0.752
FANK1	fibronectin type III and ankyrin repeat domains	Detected in some	61.2	16		1.060	0.097	0.719	0.147	0.928	0.105	0.371
DUSP4	dual specificity phosphatase 4	Detected in single	6.7	9		0.326	0.033	0.323	0.039	0.357	0.037	0.537
CEP55	centrosomal protein 55	Detected in single	1.3	8		0.720	0.077	0.508	0.100	0.656	0.081	0.581
BOLL	boule homolog, RNA binding protein	Detected in single	1.5	9		0.000		0.000		0.000		
ACTG2	actin gamma 2, smooth muscle	Detected in single	8.6	11		0.023	0.014	0.038	0.021	0.033	0.019	0.660
HACD1	3-hydroxyacyl-CoA dehydratase 1	Detected in many	55.7	11		1.291	0.082	1.119	0.142	1.174	0.109	0.389
					Average	1.084		0.929		1.003		
					T test			0.0319664	Paired T	0.188032		

T cells, gamma delta						n=38		n=19		n=25		Low vs
						LOW	LOW	MID		CAD		CAD
						Mean	s.e.m.	Mean	s.e.m.	Mean	s.e.m.	T test
Gene	Description	Cell lineage d	nTPM	Specificity score								
ACTG2	actin gamma 2, smooth muscle	Detected in si	8.6	11		0.023	0.014	0.038	0.021	0.033	0.019	0.66
BOLL	boule homolog, RNA binding protein	Detected in si	1.5	9		0.000	0.000	0.000	0.000	0.000	0.000	#DIV/0!
CEP55	centrosomal protein 55	Detected in si	1.3	8		0.720	0.077	0.508	0.100	0.656	0.081	0.581
DUSP4	dual specificity phosphatase 4	Detected in si	6.7	9		0.326	0.033	0.323	0.039	0.357	0.037	0.537
FANK1	fibronectin type III and ankyrin repeat domain	Detected in sc	61.2	16		1.060	0.097	0.719	0.147	0.928	0.105	0.371
FOXP3	forkhead box P3	Detected in sc	53.3	11		2.489	0.240	2.378	0.342	2.621	0.361	0.752
HACD1	3-hydroxyacyl-CoA dehydratase 1	Detected in m	55.7	11		1.291	0.082	1.119	0.142	1.174	0.109	0.389
OGN	osteoglycin	Detected in si	1.7	9		0.117	0.020	0.105	0.028	0.134	0.035	0.653
PMCH	pro-melanin concentrating hormone	Detected in sc	9.5	10		0.051	0.025	0.031	0.031	0.000	0.000	0.104
RTKN2	rhotekin 2	Detected in sc	16.7	8		3.944	0.362	3.183	0.447	3.268	0.449	0.245
SEMA3G	semaphorin 3G	Detected in si	1.4	15		0.398	0.034	0.289	0.046	0.35	0.031	0.333
TRIM16	tripartite motif containing 16	Detected in m	45.1	9		2.592	0.191	2.458	0.28	2.513	0.235	0.796
					Average	1.08425		0.92925		1.0028333		
					T test			0.0317477		0.187311		

T cells, MAIT						n=38		n=19		n=25		Low vs
						LOW	LOW	MID		CAD		CAD
						Mean	s.e.m.	Mean	s.e.m.	Mean	s.e.m.	T test
Gene	Description	Cell type distribution	nTPM	Specificity score								
NTN4	netrin 4	Detected in single	1.6	12		0.451	0.058	0.302	0.072	0.334	0.065	0.193
PRSS35	serine protease 35	Detected in some	19.5	9		0.461	0.097	0.209	0.055	0.259	0.076	0.136
TPBG	trophoblast glycoprotein	Detected in single	1.4	8		0	0	0	0	0	0	#DIV/0!
SLC4A10	solute carrier family 4 member 10	Detected in some	63.3	8		3.526	0.526	1.967	0.526	2.102	0.294	0.044
ITPKA	inositol-trisphosphate 3-kinase A	Detected in single	1.4	8		0.058	0.015	0.104	0.041	0.053	0.017	0.848
IL23R	interleukin 23 receptor	Detected in some	21.7	8		1.026	0.138	0.633	0.152	0.532	0.096	0.011
ME1	malic enzyme 1	Detected in some	16	7		1.181	0.098	0.892	0.114	1.278	0.121	0.537
TSPAN15	tetraspanin 15	Detected in many	19.5	6		1.421	0.126	1.366	0.205	1.491	0.216	0.768
ELOVL4	ELOVL fatty acid elongase 4	Detected in some	23.7	6		1.198	0.119	0.771	0.157	0.919	0.139	0.136
CYP27B1	cytochrome P450 family 27 subfamily B m	Detected in single	1.2	6		0.147	0.029	0.116	0.039	0.089	0.028	0.18
ROR2	receptor tyrosine kinase like orphan recep	Detected in single	1	6		0.082	0.017	0.08	0.033	0.073	0.025	0.75
RORC	RAR related orphan receptor C	Detected in many	50.3	6		3.874	0.379	2.691	0.461	2.674	0.362	0.034
					Average	1.11875		0.7609167		0.817		
					T test			0.0319906		0.0613481		

B-Cells						n=38		n=19		n=25		Low vs
						LOW	LOW	MID		CAD		CAD
						Mean	s.e.m.	Mean	s.e.m.	Mean	s.e.m.	T test
**Gene**	**Description**	**Cell type dist**	**nTPM**	**Specificity score**								
VPREB3	V-set pre-B cell surrogate l	Detected in n	803.7	732		9.606	1.068	7.383	1.414	9.094	1.678	0.788
AC233755.1	immunoglobulin heavy vari	Detected in s	403	447		0	0	0	0	0	0	0
AC233755.2		Detected in s	34.3	344		0	0	0	0	0	0	0
COL19A1	collagen type XIX alpha 1 ch	Detected in s	31.9	299		4.235	0.392	3.845	0.78	5.05	0.887	0.35
IGLL5	immunoglobulin lambda lik	Detected in n	615.5	246		47.967	10.46	27.434	5.076	34.558	5.396	0.33
AC141272.1		Detected in s	17.6	176		0	0	0	0	0	0	0
AC136616.2		Detected in s	21.7	150		0	0	0	0	0	0	0
WNT16	Wnt family member 16	Detected in s	15.6	146		0.712	0.122	0.59	0.136	0.649	0.114	0.722
KLHL14	kelch like family member 1	Detected in s	10.5	106		2.618	0.25	2.092	0.313	2.891	0.552	0.617
CPNE5	copine 5	Detected in s	51.6	105		4.831	0.376	4.043	0.455	4.82	0.362	0.984
MS4A1	membrane spanning 4-dom	Detected in n	627	96		64.988	5.706	52.272	6.659	69.473	10.637	0.688
CD19	CD19 molecule	Detected in n	172.1	89		11.709	1.119	9.076	1.233	13.043	2.059	0.54
					Average	12.2221667		8.89458333		11.6315		
					T test			0.10335869		0.63949269		

B-cells Naïve						n=38		n=19		n=25		Low vs
						LOW	LOW	MID		CAD		CAD
						Mean	s.e.m.	Mean	s.e.m.	Mean	s.e.m.	T test
**Gene**	**Description**	**Cell type dist**	**nTPM**	**Specificity score**								
KCNG1	potassium voltage-gated ch	Detected in s	7.1	20		0.847	0.121	0.703	0.177	1.268	0.217	0.072
GPR17	G protein-coupled receptor	Detected in s	1.9	19		0.137	0.022	0.101	0.027	0.105	0.023	0.34
APBB2	amyloid beta precursor prot	Detected in s	5.6	14		0.259	0.031	0.244	0.041	0.321	0.063	0.34
DSP	desmoplakin	Detected in s	2	13		1.409	0.297	1.121	0.316	1.382	0.285	0.951
AC136616.3		Detected in s	5	11		0	0	0	0	0	0	0
JSRP1	junctional sarcoplasmic reti	Detected in s	2.6	10		0.287	0.049	0.209	0.055	0.332	0.078	0.609
SLC26A4	solute carrier family 26 mer	Detected in s	1	8		0.1	0.014	0.047	0.019	0.188	0.033	0.007
TUBB3	tubulin beta 3 class III	Detected in s	2.5	8		0.122	0.028	0.123	0.045	0.084	0.027	0.362
CCN2	cellular communication net	Detected in s	8.1	7		0.158	0.043	0.214	0.055	0.148	0.044	0.88
AKAP6	A-kinase anchoring protein	Detected in s	3.5	6		0.646	0.061	0.747	0.164	0.73	0.107	0.468
SLC2A5	solute carrier family 2 mem	Detected in s	23.3	5		0.826	0.079	0.794	0.098	0.944	0.119	0.39
H4C4	H4 clustered histone 4	Detected in s	3.4	4		42.323	2.583	38.367	4.064	37.35	2.364	0.186
					Average	3.92616667		3.55583333		3.571		
					T test			0.28179011		0.41719726		

B cells, Memory						n=38		n=19		n=25		Low vs
						LOW	LOW	MID		CAD		CAD
						Mean	s.e.m.	Mean	s.e.m.	Mean	s.e.m.	T test
**Gene**	**Description**	**Cell type dist**	**nTPM**	**Specificity score**								
TAS1R3	taste 1 receptor member 3	Detected in s	1.6	17		0.736	0.07	0.673	0.124	0.627	0.058	0.274
ARL14	ADP ribosylation factor like	Detected in s	1.8	15		0.048	0.019	0	0	0.022	0.016	0.325
APOD	apolipoprotein D	Detected in s	5	14		0.026	0.015	0.425	0.221	0.086	0.049	0.18
RASSF6	Ras association domain far	Detected in s	2.7	8		0.433	0.047	0.301	0.087	0.325	0.044	0.118
IL5	interleukin 5	Detected in s	1.4	8		0	0	0	0	0	0	#DIV/0!
DKKL1	dickkopf like acrosomal pro	Detected in s	1.7	6		0.008	0.008	0.051	0.028	0.028	0.02	0.312
H3C7	H3 clustered histone 7	Detected in s	1.8	5		18.596	1.371	14.927	1.683	17.102	1.187	0.446
COL4A4	collagen type IV alpha 4 cha	Detected in s	4.1	5		0.765	0.076	0.617	0.093	0.729	0.144	0.811
SPACA3	sperm acrosome associate	Detected in s	1.2	5		0.033	0.021	0	0	0.05	0.036	0.674
PXDC1	PX domain containing 1	Detected in s	1.2	4		1.304	0.107	1.33	0.253	1.284	0.134	0.909
SLC16A4	solute carrier family 16 me	Detected in s	1.5	4		0.718	0.064	0.658	0.095	0.632	0.058	0.348
NPIPB15	nuclear pore complex intera	Detected in s	1	4		1.805	0.281	1.547	0.38	1.972	0.307	0.698
					Average	2.03933333		1.71075		1.90475		
					T test			0.3074733		0.3066404		

NK Cells						n=38		n=19		n=25		Low vs
						LOW	LOW	MID		CAD		CAD
						Mean	s.e.m.	Mean	s.e.m.	Mean	s.e.m.	T test
**Gene**	**Description**	**Cell type distri**	**nTPM**	**Specificity score**								
COX6A2	cytochrome c oxidase sub	Detected in sin	15	150		0	0	0	0	0	0	#DIV/0!
ZMAT4	zinc finger matrin-type 4	Detected in sin	8.6	86		0.412	0.085	0.4	0.116	0.352	0.074	0.625
APOC2	apolipoprotein C2	Detected in sin	3.2	30		0	0	0	0	0	0	#DIV/0!
KIR2DL4	killer cell immunoglobuli	Detected in sin	2.8	28		0	0	0	0	0	0	#DIV/0!
BAALC	BAALC binder of MAP3K1	Detected in sin	3.7	28		0.193	0.037	0.128	0.03	0.221	0.042	0.616
EXOC1L	exocyst complex compon	Detected in sin	1.9	20		0	0	0.022	0.022	0	0	#DIV/0!
NFILZ	NFIL3 like basic leucine z	Detected in sin	17.5	20		0	0	0.022	0.022	0	0	#DIV/0!
SERPINE1	serpin family E member	Detected in sin	9.5	20		1.545	0.142	1.19	0.159	1.944	0.335	0.221
RAMP1	receptor activity modifyir	Detected in sol	70.1	18		0.811	0.119	0.905	0.195	1.221	0.185	0.055
TRIM54	tripartite motif containin	Detected in sin	3.6	17		0.011	0.007	0.018	0.018	0.004	0.004	0.539
LGALS9B	galectin 9B	Detected in sin	12.7	17		0.907	0.127	0.45	0.121	0.787	0.177	0.574
PRDM16	PR/SET domain 16	Detected in sin	2.4	16		0.021	0.006	0.027	0.008	0.016	0.006	0.561
					Average	0.325		0.2635		0.37875		
					T test			0.22618379		0.29213006		

Dendritic Cells						n=38		n=19		n=25		Low vs
						LOW	LOW	MID		CAD		CAD
						Mean	s.e.m.	Mean	s.e.m.	Mean	s.e.m.	T test
**Gene**	**Description**	**Cell type dist**	**nTPM**	**Specificity score**								
LRRC26	leucine rich repeat containi	Detected in s	117.6	1177		0.047	0.016	0.043	0.025	0.088	0.034	0.234
TPM2	tropomyosin 2	Detected in s	398.6	920		6.743	0.593	5.995	0.795	6.347	0.648	0.662
ASIP	agouti signaling protein	Detected in s	134.8	356		0.097	0.036	0.11	0.068	0.091	0.045	0.92
KRT5	keratin 5	Detected in s	32.6	326		0.424	0.099	0.309	0.085	0.23	0.043	0.133
TNFRSF21	TNF receptor superfamily m	Detected in s	69.2	303		1.098	0.099	1.138	0.154	0.814	0.094	0.052
GPM6B	glycoprotein M6B	Detected in s	77.7	268		0.111	0.015	0.09	0.026	0.087	0.017	0.291
SCARA5	scavenger receptor class A	Detected in s	21.1	211		0.146	0.024	0.204	0.054	0.175	0.05	0.575
SHD	Src homology 2 domain con	Detected in s	17.8	178		0.167	0.037	0.255	0.086	0.199	0.052	0.609
NTM	neurotrimin	Detected in s	17.4	175		0.01	0.005	0.005	0.004	0.012	0.008	0.826
SCT	secretin	Detected in s	126.7	138		0	0	0	0	0	0	#DIV/0!
KCNA5	potassium voltage-gated ch	Detected in s	12.2	122		0.154	0.02	0.092	0.026	0.055	0.019	0.001
SERPINF1	serpin family F member 1	Detected in a	1454.4	121		2.893	0.218	2.543	0.381	2.395	0.234	0.137
					Average	0.99083333		0.89866667		0.87441667		
					T test			0.20251312		0.0513431		

**Myeloid Dendritic Cells**						n=38		n=19		n=25		Low vs
						LOW	LOW	MID		CAD		CAD
						Mean	s.e.m.	Mean	s.e.m.	Mean	s.e.m.	T test
**Gene**	**Description**	**Cell type dist**	**nTPM**	**Specificity score**								
MRC1	mannose receptor C-type 1	Detected in s	4.8	16		0.939	0.105	0.812	0.133	0.927	0.126	0.941
CRIP3	cysteine rich protein 3	Detected in n	44.6	16		4.156	0.351	3.2	0.347	3.582	0.373	0.281
CD1E	CD1e molecule	Detected in s	62.5	14		0.834	0.1	0.666	0.191	0.809	0.122	0.874
HTR7	5-hydroxytryptamine recept	Detected in s	1.4	12		0.581	0.054	0.571	0.131	0.531	0.076	0.585
CD207	CD207 molecule	Detected in s	1.1	12		0.251	0.048	0.135	0.039	0.214	0.047	0.591
CLEC10A	C-type lectin domain contai	Detected in n	465.9	12		17.477	1.419	12.356	1.764	12.296	1.028	0.01
PKIB	cAMP-dependent protein ki	Detected in s	34.8	11		0.516	0.04	0.41	0.115	0.537	0.073	0.791
NDRG2	NDRG family member 2	Detected in n	204.4	11		2.527	0.158	2.168	0.268	1.946	0.243	0.04
DEPTOR	DEP domain containing MT(	Detected in s	20.3	11		1.06	0.088	0.876	0.126	0.735	0.105	0.022
EHF	ETS homologous factor	Detected in s	1.7	11		0.239	0.033	0.099	0.025	0.189	0.04	0.345
C19orf33	chromosome 19 open readi	Detected in n	22.2	10		4.726	0.614	4.242	1.226	5.015	0.877	0.782
SDS	serine dehydratase	Detected in s	11.6	10		0.183	0.036	0.079	0.036	0.232	0.057	0.439
					Average	2.79075		2.1345		2.25108333		
					T test			0.1399894		0.23373625		

**Plasmacytoid Dendritic Cells**						n=38		n=19		n=25		Low vs
						LOW	LOW	MID		CAD		CAD
						Mean	s.e.m.	Mean	s.e.m.	Mean	s.e.m.	T test
**Gene**	**Description**	**Cell type dist**	**nTPM**	**Specificity score**								
LRRC26	leucine rich repeat containi	Detected in s	117.6	1177		0.047	0.016	0.043	0.025	0.088	0.034	0.234
TPM2	tropomyosin 2	Detected in s	398.6	812		6.743	0.593	5.995	0.795	6.347	0.648	0.662
ASIP	agouti signaling protein	Detected in s	134.8	356		0.097	0.036	0.11	0.068	0.091	0.045	0.92
KRT5	keratin 5	Detected in s	32.6	326		0.424	0.099	0.309	0.085	0.23	0.043	0.133
GPM6B	glycoprotein M6B	Detected in s	77.7	220		0.111	0.015	0.09	0.026	0.087	0.017	0.291
SHD	Src homology 2 domain con	Detected in s	17.8	178		0.167	0.037	0.255	0.086	0.199	0.052	0.609
NTM	neurotrimin	Detected in s	17.4	175		0.01	0.005	0.005	0.004	0.012	0.008	0.826
SCT	secretin	Detected in s	126.7	138		0	0	0	0	0	0	#DIV/0!
KCNA5	potassium voltage-gated ch	Detected in s	12.2	122		0.154	0.02	0.092	0.026	0.055	0.019	0.001
SCARA5	scavenger receptor class A	Detected in s	21.1	121		0.146	0.024	0.204	0.054	0.175	0.05	0.575
MYMX	myomixer, myoblast fusion	Detected in s	10.6	106		0.06	0.027	0.035	0.024	0.012	0.012	0.17
EPHA2	EPH receptor A2	Detected in s	9.4	94		0.328	0.034	0.303	0.068	0.222	0.041	0.05
					Average	0.69058333		0.62008333		0.6265		
					T test			0.28957847		0.10408841		

**Granulocytes**						n=38		n=19		n=25		Low vs
						LOW	LOW	MID		CAD		CAD
						Mean	s.e.m.	Mean	s.e.m.	Mean	s.e.m.	T test
**Gene**	**Description**	**Cell type dist**	**nTPM**	**Specificity score**								
CPA3	carboxypeptidase A3	Detected in s	1115.4	5518		7.595	0.657	6.164	0.699	5.796	0.711	0.075
MS4A3	membrane spanning 4-do	Detected in s	2686.3	4507		0.931	0.116	0.852	0.136	1.151	0.176	0.28
PI3	peptidase inhibitor 3	Detected in s	1763.3	4377		50.881	5.887	64.506	14.126	83.671	13.757	0.016
GATA2	GATA binding protein 2	Detected in s	513.2	4344		11.189	0.869	11.198	1.531	10.662	1.447	0.741
CLC	Charcot-Leyden crystal gal	Detected in a	28756.2	2254		184.127	29.421	160.17	30.322	192.862	25.04	0.834
HDC	histidine decarboxylase	Detected in s	686.2	2140		11.623	0.999	10.802	1.382	10.612	1.682	0.584
GATA1	GATA binding protein 1	Detected in s	471.7	1877		25.446	1.305	27.785	2.046	28.133	2.14	0.26
TCN1	transcobalamin 1	Detected in n	2374.2	1592		7.553	0.884	7.391	0.838	9.015	2.009	0.458
RHEX	regulator of hemoglobiniz	Detected in s	1312.1	1544		3.552	0.217	3.1	0.456	3.571	0.403	0.964
MS4A2	membrane spanning 4-do	Detected in s	275.5	1467		4.547	0.405	4.737	0.797	4.356	0.613	0.787
CA4	carbonic anhydrase 4	Detected in s	99.5	995		7.308	0.814	9.1	1.526	8.731	1.397	0.35
TPSAB1	tryptase alpha/beta 1	Detected in s	89.3	893		0.076	0.023	0.091	0.033	0.182	0.053	0.044
					Average	26.2356667		25.4913333		29.8951667		
					T test			0.76289676		0.21201408		

Neutrophils						n=38		n=19		n=25		Low vs
						LOW	LOW	MID		CAD		CAD
						Mean	s.e.m.	Mean	s.e.m.	Mean	s.e.m.	T test
Gene	Description	Cell type dist	nTPM	Specificity score								
PI3	peptidase inhibitor 3	Detected in s	1763.3	1021		50.881	5.887	64.506	14.126	83.671	13.757	0.016
CHI3L1	chitinase 3 like 1	Detected in s	470.4	495		141.947	16.424	139.227	19.69	124.216	18.235	0.482
CXCL8	C-X-C motif chemokine liga	Detected in s	483	451		14.528	1.533	17.599	2.216	13.975	1.332	0.801
ANXA3	annexin A3	Detected in s	207.7	320		66.237	12.112	72.68	17.208	95.174	27.213	0.281
TGM3	transglutaminase 3	Detected in s	30.3	295		3.484	0.354	3.802	0.384	3.904	0.463	0.468
CXCL1	C-X-C motif chemokine liga	Detected in s	47.3	243		14.857	0.998	16.194	1.404	14.735	1.273	0.94
BTNL3	butyrophilin like 3	Detected in s	22.3	206		4.045	1.222	5.151	1.393	6.059	1.372	0.286
C4BPA	complement component 4	Detected in s	29.5	137		5.069	1.473	7	5.674	5.06	1.872	0.997
MMP9	matrix metallopeptidase 9	Detected in s	272.2	127		85.885	15.088	93.372	18.794	107.173	21.973	0.412
MME	membrane metalloendope	Detected in s	139.4	122		145.13	11.224	138.011	9.494	154.052	15.645	0.636
CXCL6	C-X-C motif chemokine liga	Detected in s	10.4	104		1.225	0.101	1.032	0.169	1.266	0.164	0.82
OTX1	orthodenticle homeobox 1	Detected in s	9.4	95		1.283	0.17	1.261	0.228	1.476	0.183	0.454
					Average	44.5475833		46.6529167		50.89675		
					T test			0.19308914		0.15486374		

Eosinophils						n=38		n=19		n=25		Low vs
						LOW	LOW	MID		CAD		CAD
						Mean	s.e.m.	Mean	s.e.m.	Mean	s.e.m.	T test
Gene	Description	Cell type dist	nTPM	Specificity score								
SEMG1	semenogelin 1	Detected in s	26.8	239		0.796	0.111	0.814	0.198	0.964	0.121	0.325
CEBPE	CCAAT enhancer binding pr	Detected in n	750.4	218		6.397	1.186	5.333	0.813	6.686	0.787	0.857
ALOX15	arachidonate 15-lipoxygena	Detected in s	602.9	193		18.593	5.519	14.68	3.839	17.003	2.835	0.826
CCL23	C-C motif chemokine ligand	Detected in s	183.7	163		0.204	0.069	0.128	0.055	0.205	0.063	0.993
PRSS41	serine protease 41	Detected in s	10.5	105		0.788	0.201	0.831	0.246	0.994	0.168	0.47
LRRC17	leucine rich repeat containi	Detected in s	9.9	94		0.332	0.054	0.278	0.076	0.328	0.047	0.964
PRSS33	serine protease 33	Detected in s	1296.1	65		8.624	2.298	8.219	2.234	8.662	1.331	0.99
THBS4	thrombospondin 4	Detected in s	4.5	46		0.713	0.137	0.502	0.112	0.763	0.113	0.797
TMIGD3	transmembrane and immu	Detected in s	62.8	44		1.233	0.279	1.662	0.76	1.508	0.443	0.581
ADORA3	adenosine A3 receptor	Detected in n	601.6	40		3.881	0.664	3.83	0.608	3.653	0.385	0.796
SIGLEC8	sialic acid binding Ig like lec	Detected in s	290.2	38		5.588	1.386	5.044	1.189	5.694	0.829	0.955
MYCT1	MYC target 1	Detected in s	30.7	33		2.622	0.236	2.627	0.36	2.762	0.25	0.694
					Average	4.14758333		3.66233333		4.10183333		
					T test			0.16871073		0.76011255		

Basophils						n=38		n=19		n=25		Low vs
						LOW	LOW	MID		CAD		CAD
						Mean	s.e.m.	Mean	s.e.m.	Mean	s.e.m.	T test
Gene	Description	Cell type dist	nTPM	Specificity score								
HDC	histidine decarboxylase	Detected in s	686.2	2140		11.623	0.999	10.802	1.382	10.612	1.682	0.584
CPA3	carboxypeptidase A3	Detected in s	1115.4	1534		7.595	0.657	6.164	0.699	5.796	0.711	0.075
MS4A3	membrane spanning 4-dom	Detected in s	2686.3	1478		0.931	0.116	0.852	0.136	1.151	0.176	0.28
MS4A2	membrane spanning 4-dom	Detected in s	275.5	908		4.547	0.405	4.737	0.797	4.356	0.613	0.787
TPSAB1	tryptase alpha/beta 1	Detected in s	89.3	893		0.076	0.023	0.091	0.033	0.182	0.053	0.044
IL4	interleukin 4	Detected in s	474.9	861		1.691	0.207	1.664	0.389	1.453	0.335	0.527
GCSAML	germinal center associated	Detected in s	227.7	661		2.283	0.165	2.031	0.19	2.07	0.202	0.418
TEX101	testis expressed 101	Detected in s	55.7	557		0.176	0.05	0.113	0.05	0.093	0.033	0.222
SLC45A3	solute carrier family 45 mer	Detected in s	40.5	382		5.26	0.378	4.511	0.51	5.039	0.623	0.748
CD200R1L	CD200 receptor 1 like	Detected in s	85	379		0.31	0.072	0.519	0.167	0.519	0.108	0.1
TRIM64B	tripartite motif containing 6	Detected in s	33.2	332		0.393	0.102	0.267	0.072	0.375	0.155	0.919
GATA2	GATA binding protein 2	Detected in s	513.2	330		11.189	0.869	11.198	1.531	10.662	1.447	0.741
					Average	3.8395		3.57908333		3.52566667		
					T test			0.09263095		0.08585686		

Monocytes						n=38		n=19		n=25		Low vs
						LOW	LOW	MID		CAD		CAD
						Mean	s.e.m.	Mean	s.e.m.	Mean	s.e.m.	T test
Gene	Description	Cell type dist	nTPM	Specificity score								
C1QC	complement C1q C chain	Detected in s	57.2	541		0.542	0.1	0.7	0.196	0.518	0.09	0.87
C1QB	complement C1q B chain	Detected in n	140.6	85		2.162	0.392	2.183	0.413	2.069	0.268	0.862
C1QA	complement C1q A chain	Detected in n	226.1	60		4.722	0.758	4.673	0.823	3.615	0.528	0.286
MERTK	MER proto-oncogene, tyro	Detected in s	8.6	50		3.875	0.299	3.432	0.467	3.643	0.407	0.641
PGA5	pepsinogen A5	Detected in s	4.3	44		0.052	0.019	0.061	0.039	0.021	0.015	0.251
PCDH12	protocadherin 12	Detected in s	4.6	41		0.413	0.045	0.323	0.055	0.348	0.05	0.354
C2	complement C2	Detected in s	25.9	35		0	0	0.001	0.001	0.001	0.001	0.432
ALDH1A1	aldehyde dehydrogenase 1	Detected in n	87.3	33		22.454	1.631	18.82	2.206	20.254	2	0.398
AVPR2	arginine vasopressin recep	Detected in s	3.6	29		0.134	0.03	0.101	0.036	0.134	0.034	0.989
PGA4	pepsinogen A4	Detected in s	10.9	26		0	0	0	0	0	0	#DIV/0!
SASH1	SAM and SH3 domain cont	Detected in s	2.6	26		2.814	0.219	3.205	0.498	2.922	0.3	0.768
CTTNBP2	cortactin binding protein 2	Detected in s	7.7	26		0.228	0.025	0.19	0.075	0.206	0.033	0.597
					Average	3.11633333		2.80741667		2.81091667		
					T test			0.33606165		0.14585008		

**Non Classical Monocytes**						n=38		n=19		n=25		Low vs
						LOW	LOW	MID		CAD		CAD
						Mean	s.e.m.	Mean	s.e.m.	Mean	s.e.m.	T test
**Gene**	**Description**	**Cell type dist**	**nTPM**	**Specificity score**								
LYPD2	LY6/PLAUR domain containing 2	Detected in s	440.1	13		3.398	0.392	1.993	0.408	3.405	0.786	0.994
L1TD1	LINE1 type transposase domain conta	Detected in s	5	13		0.825	0.124	0.467	0.092	0.482	0.112	0.059
CKB	creatine kinase B	Detected in r	290.8	10		2.259	0.189	1.958	0.285	2.297	0.204	0.896
CTTNBP2	cortactin binding protein 2	Detected in s	7.7	9		0.228	0.025	0.19	0.075	0.206	0.033	0.597
VMO1	vitelline membrane outer layer 1 hom	Detected in s	111.9	9		1.776	0.334	1.138	0.487	1.106	0.208	0.138
PAQR4	progestin and adipoQ receptor family	Detected in s	3.6	8		3.14	0.205	2.337	0.27	2.981	0.263	0.634
PCDH12	protocadherin 12	Detected in s	4.6	8		0.413	0.045	0.323	0.055	0.348	0.05	0.354
THAP10	THAP domain containing 10	Detected in s	4.2	7		0.115	0.021	0.212	0.043	0.144	0.03	0.412
MRAS	muscle RAS oncogene homolog	Detected in s	25	6		1.793	0.158	1.317	0.227	1.441	0.169	0.146
GSTA4	glutathione S-transferase alpha 4	Detected in r	20	6		1.317	0.133	1.256	0.187	1.159	0.141	0.431
ICAM4	intercellular adhesion molecule 4 (La	Detected in r	215.4	6		5.793	0.351	4.46	0.427	4.768	0.39	0.06
PPM1N	protein phosphatase, Mg2+/Mn2+ de	Detected in a	142.4	6		2.027	0.131	1.61	0.193	2.009	0.187	0.937
					Average	1.92366667		1.43841667		1.6955		
					T test			0.00547687		0.03404202		

**Classical Monocytes**						n=38		n=19		n=25		Low vs
						LOW	LOW	MID		CAD		CAD
						Mean	s.e.m.	Mean	s.e.m.	Mean	s.e.m.	T test
**Gene**	**Description**	**Cell type dist**	**nTPM**	**Specificity score**								
S1PR3	sphingosine-1-phosphate re	Detected in s	7	18		6.031	0.424	6.367	0.859	5.874	0.574	0.824
RXFP2	relaxin family peptide recep	Detected in s	1.7	10		0.004	0.003	0.004	0.004	0	0	0.289
CLEC5A	C-type lectin domain contai	Detected in s	9.1	8		2.891	0.186	3.216	0.691	3.271	0.435	0.369
CYP1B1	cytochrome P450 family 1 s	Detected in n	156.9	7		26.163	2.057	26.562	2.656	28.64	3.724	0.532
TDRD9	tudor domain containing 9	Detected in s	1.6	6		3.572	0.321	4.492	1.16	4.05	0.623	0.46
ALDH1A1	aldehyde dehydrogenase 1	Detected in n	87.3	6		22.454	1.631	18.82	2.206	20.254	2	0.398
CCL8	C-C motif chemokine ligand	Detected in s	3.4	6		0.163	0.042	0.124	0.039	0.215	0.064	0.479
ABCC6	ATP binding cassette subfa	Detected in s	3.4	6		0.15	0.013	0.137	0.026	0.131	0.018	0.378
NRG1	neuregulin 1	Detected in s	55.3	5		1.532	0.163	1.426	0.194	1.586	0.202	0.834
FXYD6	FXYD domain containing ioi	Detected in s	37.7	4		1.731	0.141	2.039	0.288	1.765	0.165	0.877
					Average	6.4691		6.3187		6.5786		
					T test			0.71479533		0.76393477		
